# Comparison of Dimethyl Fumarate vs Fingolimod and Rituximab vs Natalizumab for Treatment of Multiple Sclerosis

**DOI:** 10.1001/jamanetworkopen.2021.34627

**Published:** 2021-11-16

**Authors:** Jue Hou, Nicole Kim, Tianrun Cai, Kumar Dahal, Howard Weiner, Tanuja Chitnis, Tianxi Cai, Zongqi Xia

**Affiliations:** 1 Department of Biostatistics, Harvard T.H. Chan School of Public Health, Boston, Massachusetts; 2 Division of Rheumatology, Department of Medicine, Brigham and Women’s Hospital, Boston, Massachusetts; 3 Department of Neurology, Brigham and Women’s Hospital, Boston, Massachusetts; 4 Department of Biomedical Informatics, Harvard Medical School, Boston, Massachusetts; 5 Department of Neurology, University of Pittsburgh, Pittsburgh, Pennsylvania; 6 Department of Biomedical Informatics, University of Pittsburgh, Pittsburgh, Pennsylvania

## Abstract

**Importance:**

As disease-modifying treatment options for multiple sclerosis increase, comparisons of the options based on real-world evidence may guide clinical decision-making.

**Objective:**

To compare the relapse outcomes between 2 pairs of disease-modifying treatments: dimethyl fumarate vs fingolimod and natalizumab vs rituximab.

**Design, Setting, and Participants:**

This comparative effectiveness study integrated data from a clinic-based multiple sclerosis research registry and its linked electronic health records (EHR) system between January 1, 2006, and December 31, 2016, and built treatment groups for each pairwise disease-modifying treatment comparison according to both registry records and electronic prescriptions. Parallel analyses were conducted from October 11, 2019, to July 7, 2021.

**Main Outcomes and Measures:**

The main outcomes were the 1-year and 2-year relapse rates as well as the time to relapse. To compare relapse outcomes, the study adjusted for covariates from 2 sources (registry and EHR) and corrected for confounding biases among the covariates by the doubly robust estimation.

**Results:**

The study included 4 treatment groups: dimethyl fumarate (n = 260; 198 women [76.2%]; 227 non-Hispanic White individuals [87.3%]; mean [SD] age at diagnosis, 41.7 [10.4] years), fingolimod (n = 267; 190 women [71.2%]; 222 non-Hispanic White individuals [83.1%]; mean [SD] age at diagnosis, 37.9 [9.9] years), natalizumab (n = 204; 160 women [78.4%]; 172 non-Hispanic White individuals [84.3%]; mean [SD] age at diagnosis, 37.2 [10.6] years), and rituximab (n = 115; 83 women [72.2%]; 99 non-Hispanic White individuals [86.1%]; mean [SD] age at diagnosis, 44.1 [11.1] years). No significant differences were found in the relapse outcomes between dimethyl fumarate and fingolimod after correcting for confounding biases and multiple testing (difference in 1-year relapse rate, 0.028 [95% CI, –0.031 to 0.084]; difference in 2-year relapse rate, 0.071 [95% CI, 0.008-0.128]; relative risk of 2-year non-relapse, 0.957 [95% CI, 0.884-1.035] with dimethyl fumarate as reference). When compared with rituximab, natalizumab was associated with a higher relapse rate for all 3 outcomes after bias correction and multiple testing (difference in 1-year relapse rate, 0.080 [95% CI, 0.013-0.137]; difference in 2-year relapse rate, 0.132 [95% CI, 0.043-0.189]; relative risk of 2-year non-relapse, 0.903 [95% CI, 0.822-0.944]). Confounders were identified from EHR data not recorded in the registry data through data-driven feature selection.

**Conclusions and Relevance:**

This study reports real-world evidence of equivalent relapse outcomes between dimethyl fumarate and fingolimod and relapse reduction in favor of rituximab relative to natalizumab. This approach illustrates the value of incorporating EHR data as high-dimensional covariates in real-world treatment comparison.

## Key Points

**Question:**

In the management of multiple sclerosis, is there a difference in relapse outcomes associated with commonly prescribed, standard-efficacy medications as well as with common higher-efficacy medications?

**Findings:**

This comparative effectiveness study integrated electronic health records with research registry data and found no significant differences in relapse outcomes between dimethyl fumarate and fingolimod after correcting for confounding biases. Rituximab was associated with a lower relapse rate when compared with natalizumab after bias correction.

**Meaning:**

The study illustrates the value of incorporating electronic health record data as high-dimensional covariates in real-world comparative effectiveness analysis of multiple sclerosis medications.

## Teaser

This comparative effectiveness study uses data from a multiple sclerosis registry and linked electronic health records to compare the relapse outcomes between 2 disease-modifying treatment pairs: dimethyl fumarate vs fingolimod and natalizumab vs rituximab.

## Introduction

The multiple sclerosis (MS) treatment landscape has changed considerably because of the growing number of approved disease-modifying therapies (DMTs).^1,2^ Standard-efficacy DMTs (eg, dimethyl fumarate and fingolimod) and higher-efficacy DMTs (eg, natalizumab and rituximab) are commonly prescribed DMTs in the United States, but, to our knowledge, there is no randomized clinical trial and limited real-world evidence for head-to-head comparison between these DMT pairs.^3^

Prior observational studies comparing dimethyl fumarate and fingolimod mostly reported similar clinical outcomes,^4,5,6,7,8^ although 1 study reported a better relapse rate associated with fingolimod.^9^ Prior studies comparing natalizumab^10,11,12,13,14,15^ and rituximab^16,17,18^ with standard-efficacy DMTs (eg, dimethyl fumarate and fingolimod) reported both natalizumab and rituximab as associated with better relapse outcomes. However, studies directly comparing rituximab and natalizumab yielded mixed results.^18,19,20^

Observational studies in MS have relied largely on research registry data.^21,22^ With advances in analytical capability,^23,24^ electronic health record (EHR) data provide unique features to complement registries. Our group previously integrated registry data from a well-characterized, longitudinal clinic-based cohort with EHR data for developing models to classify MS diagnosis and severity,^25^ assessing comorbidity burden,^26^ examining long-term disease activity trends,^27^ and predicting future relapse.^28^ Here, we compared 2 DMT pairs using registry-annotated MS relapse as the outcome and high-dimensional EHR features and doubly robust (DR) estimation strategies to extensively correct for confounding biases.

## Methods

### Patient Sources

The Comprehensive Longitudinal Investigation of Multiple Sclerosis (CLIMB) is a long-term study of patients with MS at Brigham and Women’s Hospital (Boston, Massachusetts).^21^ In this comparative effectiveness study, we included CLIMB participants with a neurologist-confirmed MS diagnosis who were 18 years or older and began treatment with dimethyl fumarate, fingolimod, natalizumab, or rituximab between January 1, 2006, and December 31, 2016. We obtained the EHR data for eligible participants from the Mass General Brigham (MGB; formerly the Partners) HealthCare system during the same period.^25,26^ Mass General Brigham began recording electronic prescriptions in 2005. We compared dimethyl fumarate vs fingolimod for the standard-efficacy DMT comparison and natalizumab vs rituximab for the higher-efficacy DMT comparison. The MGB institutional review board approved the use of research registry data and EHR data. CLIMB participants provided written consent. This study followed the International Society for Pharmacoeconomics and Outcomes Research (ISPOR) reporting guideline.

### DMT Data

Data on DMT exposure (ie, start and end date) were derived from 2 sources: primarily, the CLIMB registry, and secondarily, the RxNorm^29^ electronic prescription records in the MGB EHR system. Disease-modifying therapies included injectable formulations (interferon-beta [all brands] and glatiramer acetate), oral formulations (fingolimod, dimethyl fumarate, teriflunomide, mitoxantrone, and cyclophosphamide), and infusions (natalizumab, rituximab, alemtuzumab, and daclizumab). We excluded DMTs approved after the study period (eg, ocrelizumab or siponimod).

### Treatment Assignment

We excluded patients who received chemotherapy (eg, cyclophosphamide or mitoxantrone) preceding target DMT initiation. For this study, standard-efficacy DMTs included interferon-beta, glatiramer acetate, daclizumab, dimethyl fumarate, fingolimod, and teriflunomide, while higher-efficacy DMTs included natalizumab, rituximab, and alemtuzumab. The Brigham MS Center (home of the CLIMB cohort) used rituximab as a higher-efficacy DMT during the study period.

In the standard-efficacy comparison, DMT group assignment (dimethyl fumarate or fingolimod) was based on the first treatment with either drug after excluding patients who had received prior treatment with higher-efficacy DMTs. In the higher-efficacy comparison, treatment assignment (natalizumab or rituximab) was based on the common prescription pattern for this cohort during the study period. If patients first started a standard-efficacy DMT and then switched to either natalizumab or rituximab, we assigned them to the higher-efficacy DMT to which patients switched. If patients first started a higher-efficacy DMT of interest, we assigned the patient to the first ever higher-efficacy DMT.

**Figure 1.**
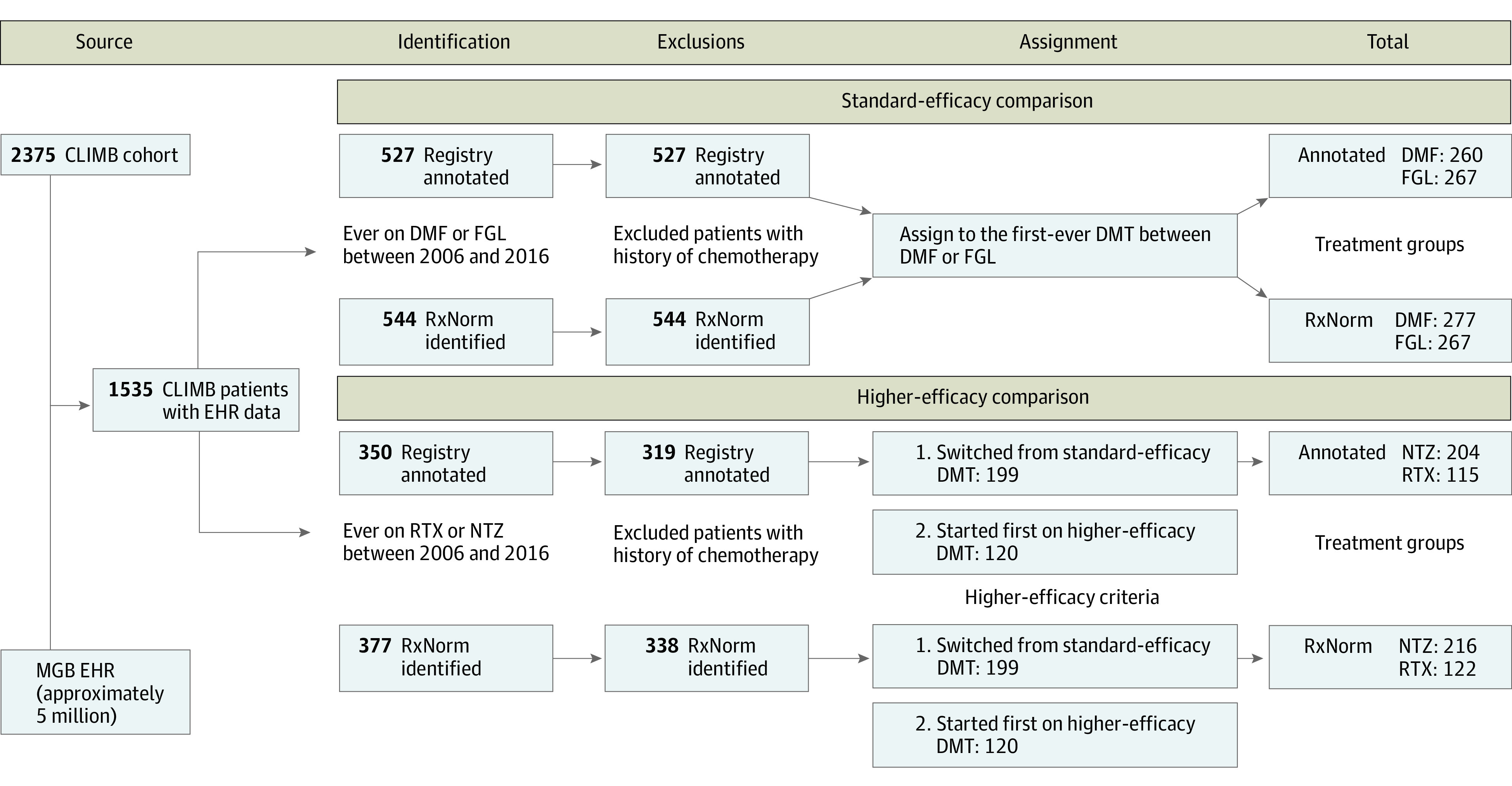
Study Schematics We linked data from the Comprehensive Longitudinal Investigation of Multiple Sclerosis at Brigham and Women’s Hospital (CLIMB) registry to the associated electronic health record (EHR) data to identify eligible patients and assign treatment groups. The available CLIMB participants with EHR data (n = 1535) represented patients with a neurologist-confirmed multiple sclerosis diagnosis who were 18 years or older (at enrollment) and began treatment with dimethyl fumarate (DMF), fingolimod (FGL), natalizumab (NTZ), or rituximab (RTX) between January 1, 2006, and December 31, 2016. Treatment groups were defined primarily based on CLIMB registry annotation and secondarily based on electronic prescriptions in the EHR (RxNorm). DMT indicates disease-modifying treatment; MGB, Mass General Brigham.

We used the CLIMB registry for “registry-annotated” treatment groups. To evaluate study feasibility beyond the registry, we performed supplementary analyses using electronic prescriptions defined by the standardized nomenclature of clinical drugs^29^ for “EHR RxNorm–identified” treatment groups. Figure 1 outlines the schematics of patient identification and treatment assignment.

### Relapse Outcomes

Relapse date and type were derived from the CLIMB registry. We defined clinical relapse as having new or recurrent neurological symptoms lasting persistently for 24 hours or longer without fever or infection. We defined radiologic relapse as having either a new T1-enhancing lesion and/or a new or enlarging T2–fluid-attenuated inversion recovery hyperintense lesion on results of brain, orbit, or spinal cord magnetic resonance imaging (MRI) studies based on clinical radiology reports. A relapse event could be a clinical and/or radiologic relapse.

For this study, we used 3 relapse outcomes: relapse rate at 1 year after treatment initiation (1-year relapse rate), relapse rate at 2 years after treatment initiation (2-year relapse rate), and the time to relapse since treatment initiation (time to relapse). The 1-year and 2-year relapse rates indicated the short-term and medium-term relapse outcomes, respectively, while the time to relapse aggregated treatment response across the follow-up duration. These relapse outcomes mirrored the typical end points in clinical trials examining the efficacy of the DMTs of interest.

### EHR Data

From the EHR, we extracted patient-level demographic and clinical information (ie, age, sex, self-reported race [American Indian or Alaska Native, Asian, Black or African American, East or Southeast Asian, South Asian, White, >1 race, or unknown or not reported] and ethnicity [Hispanic or Latino, not Hispanic or Latino, or unknown], disease duration [years elapsed between the first MS diagnostic code and index encounter at treatment initiation], and follow-up duration [years elapsed between the first of any diagnostic code and treatment initiation]).^30^ We extracted these EHR features to increase the generalizability of the method despite the availability of comparable features from the CLIMB registry.

We extracted patient-level counts of the following codified EHR features: (1) all diagnostic (*International Classification of Diseases, Ninth Revision* [*ICD-9*] and *Tenth Revision* [*ICD-10*]) codes; and (2) all procedural (*Current Procedural Terminology* [*CPT*]) codes. Using a published method to consolidate related *ICD* codes of each unique clinical condition, we mapped each *ICD* code to a single clinical condition represented by a “phenotype” code (PheCode).^24^ To mitigate sparsity, we consolidated *CPT* codes according to groupings defined by the American Medical Association except for certain MS-relevant MRI procedures (orbit, brain, and spine), which were analyzed as individual codes. We excluded laboratory values, such as JC virus antibody titer, owing to current challenges with standardizing laboratory reporting in the EHR data.

From free-text clinical narratives (eg, outpatient encounters, radiology reports, and discharge summaries), we extracted patient-level counts of all clinical terms mapped to *Concept Unique Identifiers* (*CUIs*) using the natural language processing–based clinical Text Analysis and Knowledge Extraction System (cTAKES).^31^ We included only positive mentions of *CUIs* after excluding *CUIs* with attributes of negation (eg, “no evidence of”), family history, conditional (“if, then”), or temporality (eg, future tense).

### Confounders

To account for imbalance in patient characteristics, we combined 2 sets of covariates to adequately capture confounding: (1) expert-defined features according to prior clinical knowledge and (2) high-dimensional full EHR features plus the expert-defined features. The expert-defined features included demographic (ie, age, sex, race, and ethnicity), clinical (ie, disease, follow-up, and prior DMT use duration and number of relapses in the prior 1 and 2 years), and expert-defined EHR features. Only the prior relapse history was derived from the CLIMB registry. For the expert-defined EHR features, we counted the occurrence of selected *ICD*, *CPT*, and *CUI* codes according to 3 time frames (ie, 3-month, 6-month, or overall follow-up period preceding treatment initiation), depending on the clinical context. Table 1 lists the expert-defined EHR features: (1) health care utilization measure (ie, number of all *ICD* codes and notes: 3 months and overall); (2) normalized MS *ICD* code frequency (ie, number of MS *ICD* codes [*ICD-9* code 340 or *ICD-10* code G35] divided by the number of all *ICD* codes: 3 months and overall); (3) normalized MS *CUI* frequency (ie, number of MS *CUIs* [C0026769 or C0751967] divided by health care utilization: 3 months and overall); and (4) normalized *CPT* code frequency for high-dose corticosteroid prescriptions (3 months and overall), MS-relevant MRI procedures (6 months and overall), hospitalizations (overall), and emergency department visits (overall), each defined by the number of *CPT* codes for a category divided by health care utilization within the given time frame.

**Table 1.** Characteristics of the Treatment Groups

Feature^a^	NTZ vs RTX			DMF vs FGL		
	NTZ (n = 204)	RTX (n = 115)	*P* value	DMF (n = 260)	FGL (n = 267)	*P* value
Sex, No. (%)						
Female	160 (78.4)	83 (72.2)	.26	198 (76.2)	190 (71.2)	.23
Male	44 (21.6)	32 (27.8)		62 (23.8)	77 (28.8)	
Non-Hispanic White, No. (%)	172 (84.3)	99 (86.1)	.79	227 (87.3)	222 (83.1)	.22
Age at first MS *ICD* code, mean (SD), y	37.2 (10.6)	44.1 (11.1)	<.001	41.7 (10.4)	37.9 (9.9)	<.001
Follow-up duration, mean (SD), y	3.7 (2.4)	5.1 (3.7)	.009	7.0 (1.5)	5.4 (1.9)	<.001
Disease duration, mean (SD), y	3.6 (2.4)	5.1 (3.7)	.004	6.8 (1.6)	5.4 (1.9)	<.001
Health care utilization overall, mean (SD)	5.1 (0.7)	5.0 (0.9)	.15	5.0 (0.8)	4.9 (0.7)	.04
Health care utilization within 3 mo, mean (SD)	4.5 (0.8)	3.8 (0.9)	<.001	3.3 (0.9)	3.6 (0.8)	<.001
Normalized MS *ICD* code overall, mean (SD)	0.5 (0.2)	0.5 (0.2)	.47	0.5 (0.2)	0.5 (0.1)	.002
Normalized MS *ICD* code within 3 mo, mean (SD)	0.5 (0.3)	0.5 (0.3)	.19	0.4 (0.3)	0.5 (0.3)	<.001
Normalized MS *CUI* code within 3 mo, mean (SD)	0.07 (0.07)	0.1 (0.1)	<.001	0.1 (0.1)	0.1 (0.1)	.002
Normalized corticosteroid use overall, mean (SD)	0.05 (0.05)	0.06 (0.07)	.70	0.04 (0.05)	0.05 (0.06)	.04
Normalized corticosteroid use within 3 mo, mean (SD)	0.02 (0.03)	0.02 (0.04)	.06	0.01 (0.03)	0.02 (0.04)	.001
Normalized MRI overall, mean (SD)	0.09 (0.07)	0.1 (0.09)	.03	0.2 (0.1)	0.2 (0.1)	.03
Normalized MRI within 6 mo, mean (SD)	0.04 (0.05)	0.08 (0.12)	.24	0.06 (0.08)	0.06 (0.08)	.06
Normalized hospitalization overall, mean (SD)	0.1 (0.3)	0.1 (0.3)	.91	0.1 (0.4)	0.09 (0.3)	.04
Normalized emergency department visits overall, mean (SD)	0.3 (0.4)	0.3 (0.5)	.38	0.3 (0.5)	0.3 (0.5)	.29
Months receiving prior DMT, mean (SD)	26.5 (30.8)	32.7 (37.5)	.52	43.9 (37.8)	37.0 (33.1)	.05
No. of relapses within prior 1 y, mean (SD)	1.6 (0.8)	1.3 (0.6)	<.001	1.1 (0.3)	1.3 (0.6)	<.001
No. of relapses within prior 2 y, mean (SD)	1.9 (1.1)	1.4 (0.8)	<.001	1.2 (0.5)	1.5 (1.0)	<.001

Abbreviations: *CUI*, *Concept Unique Identifier*; DMF, dimethyl fumarate; DMT, disease-modifying therapy; FGL, fingolimod; *ICD*, *International Classification of Diseases*; MRI, magnetic resonance imaging; MS, multiple sclerosis; NTZ, natalizumab; RTX, rituximab.

^a^ For electronic health record features, we counted the occurrence of selected *ICD*, *Current Procedural Terminology*, and *CUI* codes according to 3 time frames (ie, 3 months, 6 months, or overall period prior to treatment initiation).

The high-dimensional full EHR features included aggregated counts of all *ICD*, *CPT*, and *CUI* codes beyond all expert-defined features. To mitigate sparsity, we removed features with less than 10% frequency among participants. We again constructed EHR features using the 3-month and overall follow-up period prior to treatment initiation. To select relevant features, we fit marginal logistic regression models using all PheCode, *CPT*, and *CUI* occurrences within a 1-week period of an index EHR encounter, with relapse as the outcome. After applying the Benjamini-Hochberg procedure with a false discovery rate of 0.1, we removed features with insignificant *P* values.

We used the combination of expert-defined and full EHR features in the main analyses (ie, expert-defined and full EHR analysis). For benchmark, we analyzed using the unadjusted or crude data, registry-derived features (ie, registry-derived analysis), and expert-defined features alone (ie, expert-defined analysis).

### Statistical Analysis

Statistical analyses were conducted from October 11, 2019, to July 7, 2021. Because treatment decisions depended on baseline patient factors, adequate adjustment for these confounding biases was critical to infer treatment outcomes. We applied the DR estimation method involving 2 adjustments to account for treatment-by-indication biases. First, we adjusted for baseline factors by assessing their association with relapse risk along with treatments received using the outcome regression.^32^ Second, we computed the propensity score^33^ to balance the baseline factors between treatment groups through the inverse probability of treatment weighting.^34,35,36^ The outcome regression and propensity score model estimates enabled risk and confounding assessment. Finally, we combined individual-level outcome regression and propensity score adjustments in the DR estimation, which is known to be superior to either outcome regression adjustment or propensity score adjustment alone^37^ and statistically efficient.^38^ Thus, we reported the DR estimation results as the main finding.

We examined each DMT for association with 3 relapse outcomes: the 1-year and 2-year relapse rate and time to relapse after treatment initiation. For each outcome, we applied outcome regression and propensity score adjustment and calculated the DR estimation.^39,40^ We used adaptive LASSO (least absolute shrinkage and selection operator)–penalized regression to fit the outcome regression and propensity score models,^41^ a regularization approach that shrank coefficients for uninformative features to zero and simultaneously provided stable effect estimates for the informative features. We selected penalty parameters by 5-fold cross-validation and quantified estimation variability for each analysis by bootstrapping with 10 000 replicates. We adjusted for multiple testing using the bootstrap estimates (eMethods and eResults in the Supplement). We investigated unmeasured confounding by sensitivity analysis with the minimal unmeasured confounding factor (E-value) for significant associations using a published method,^42,43,44,45^ which we adapted for high-dimensional features, and by evaluation of the reduction in confounding by adjustment of full EHR features when compared with the registry-derived analysis (eMethods and eResults in the Supplement). All analyses were conducted using R, version 4.0.3 (R Group for Statstical Computing).^46^ All *P* values were from 2-sided tests, and results were deemed statistically significant at *P* < .05 after adjustment for multiple testing.

#### Data Availability

Code for analysis and figure generation is available online.^47^ Summary and anonymous data for this study are available upon reasonable request to the corresponding author.

### Supplementary and Benchmark Analyses

For comparison with the primary expert-defined and full EHR feature analysis using registry-annotated treatment groups, we conducted the following supplementary analyses: (1) unadjusted or crude analysis using registry-annotated treatment groups; (2) registry-derived feature analysis using registry-annotated groups; (3) expert-defined feature analysis using registry-annotated groups; (4) expert-defined feature analysis using EHR RxNorm–identified groups; and (5) expert-defined and full EHR feature analysis using EHR RxNorm–identified groups. To assess potential confounding owing to temporal changes in medical management, we conducted time-adjusted analyses adjusting for year of DMT initiation for natalizumab vs rituximab and matching the year of DMT initiation for dimethyl fumarate vs fingolimod.

## Results

### Patient Characteristics

For the primary analysis using registry-annotated treatment groups, we reported the baseline characteristics for eligible patients in the following groups: dimethyl fumarate (n = 260; 198 women [76.2%]; 227 non-Hispanic White individuals [87.3%]; mean [SD] age at diagnosis, 41.7 [10.4] years; mean [SD] disease duration at DMT initiation, 6.8 [1.6] years), fingolimod (n = 267; 190 women [71.2%] women; 222 non-Hispanic White individuals [83.1%]; mean [SD] age, 37.9 [9.9] years; mean [SD] disease duration, 5.4 [1.9] years), natalizumab (n = 204; 160 women [78.4%]; 172 non-Hispanic White individuals [84.3%]; mean [SD] age, 37.2 [10.6] years; mean [SD] disease duration, 3.6 [2.4] years), and rituximab (n = 115; 83 women [72.2%]; 99 non-Hispanic White individuals [86.1%]; mean [SD] age, 44.1 [11.1] years; mean [SD] disease duration, 5.1 [3.7] years) (Table 1). Inverse probability of treatment weighting balancing mostly corrected the identified confounders from high-dimensional features (eTables 1 and 2 in the Supplement).

The higher-efficacy comparison included 319 registry-annotated patients (rituximab: n = 115; natalizumab: n = 204) and 338 EHR RxNorm–identified patients (rituximab: n = 122; natalizumab: n = 216) who met the eligibility criteria. The standard-efficacy DMT comparison included 527 registry-annotated patients (dimethyl fumarate: n = 260; fingolimod: n = 267) and 544 EHR RxNorm–identified patients (dimethyl fumarate: n = 277; fingolimod: n = 267) (Figure 1). The registry-annotated and EHR RxNorm–identified treatment groups essentially overlapped.

### Natalizumab vs Rituximab

**Figure 2.**
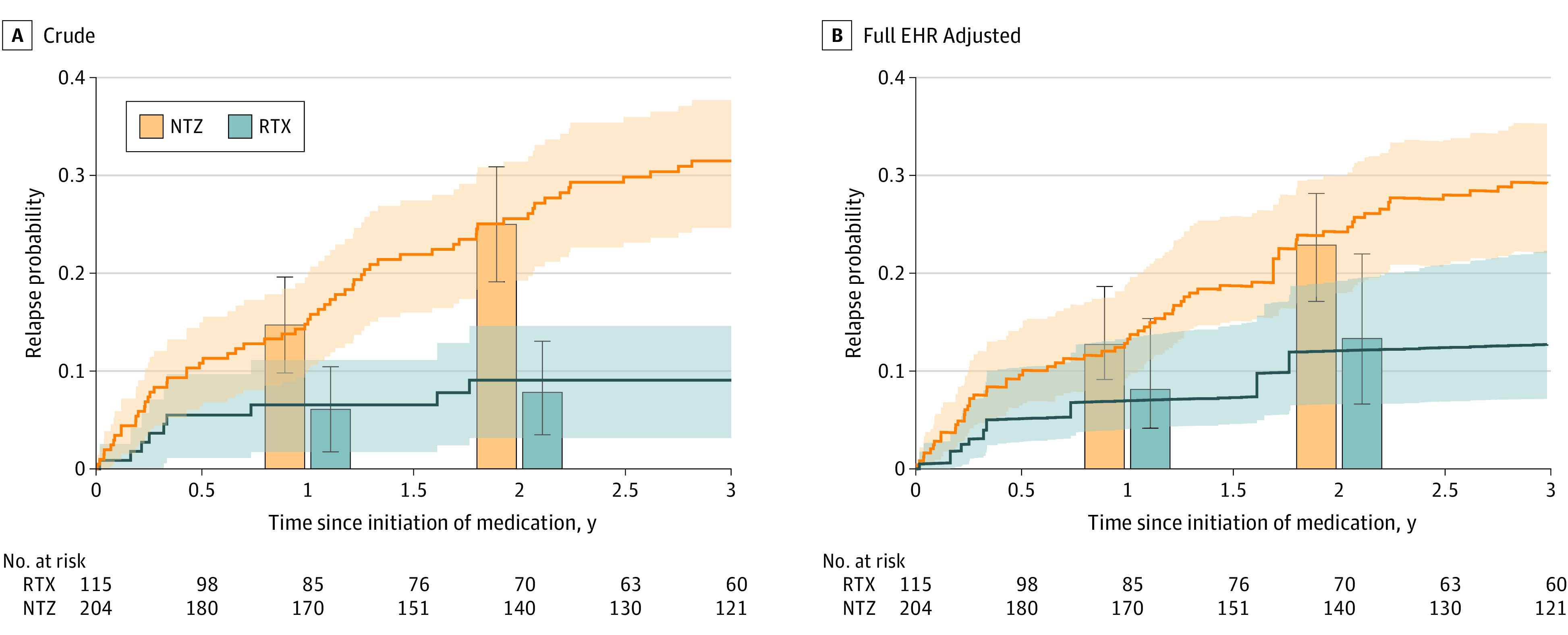
Multiple Sclerosis Relapse for Patients Treated With Natalizumab (NTZ) or Rituximab (RTX) Based on Registry-Annotated Treatment Groups The lines depict the cumulative incidence curves for time to relapse, obtained from (A) Kaplan-Meier for crude estimation and (B) mean estimated relapse probability based on doubly robust (DR) estimation with adjustment of high-dimensional full electronic health record (EHR) features in addition to expert-defined features (based on clinical knowledge). The bars indicate the relapse rates at 1 year and 2 years since treatment initiation according to crude and DR analyses. Shaded areas and error bars indicate the 95% CIs. We reported the results based on the DR estimation in Table 2.

In the primary analysis of registry-annotated treatment groups using rituximab as reference after adjusting for the high-dimensional full EHR covariates that included expert-defined features (Table 2; Figure 2), patients receiving natalizumab had a higher 1-year relapse rate (DR estimate, 0.080 [95% CI, 0.013-0.137]), higher 2-year relapse rate (DR estimate, 0.132 [95% CI, 0.043-0.189]), and shorter time to relapse (DR estimate, 0.903 [95% CI, 0.822-0.944]) than patients receiving rituximab. With rituximab as reference, a positive difference in the 1-year or 2-year relapse rate or a relative risk of non-relapse rates less than 1 indicated higher relapse probability associated with natalizumab. These consistent findings supported the association of rituximab with a lower relapse rate relative to natalizumab.

**Table 2.** Estimated Comparative Treatment Outcomes of Natalizumab vs Rituximab and Dimethyl Fumarate vs Fingolimod Based on Registry-Annotated Treatment Groups and Adjustment for Full EHR Features

Treatment	Estimate (95% CI)	*P* value^a^	E-value (E-value*)^b^
**Natalizumab vs rituximab** ^c^			
Outcome^d^			
Difference in 1-y relapse rate^e^	0.080 (0.013 to 0.137)	.02 (.02)	1.50 (1.13)
Difference in 2-y relapse rate^e^	0.132 (0.043 to 0.189)	.004 (.004)	2.26 (1.31)
Relative risk of 2-y non-relapse (from time-to-relapse analysis)^e^	0.903 (0.822 to 0.944)	<.001 (.01)	1.11 (1.06)
**Dimethyl fumarate vs fingolimod** ^f^			
Outcome^d^			
Difference in 1-y relapse rate^g^	0.028 (–0.031 to 0.084)	.38 (.38)	NA
Difference in 2-y relapse rate^g^	0.071 (0.008 to 0.128)	.03 (.08)	NA
Relative risk of 2-y non-relapse (from time-to-relapse analysis)^g^	0.957 (0.884 to 1.035)	.28 (.50)	NA

Abbreviations: EHR, electronic health record; NA, not applicable.

^a^ *P* values in parentheses are adjusted for multiple testing among the 3 analyses with the same treatment groups and feature set (eMethods and eResults in the Supplement).

^b^ E-values assess the strength of the unmeasured confounding that would change the direction of association, while E-values* assess the strength of the unmeasured confounding that would negate the significance of the observed associations. Thus, an E-value (or E-value*) indicates that residual confounding could explain the observed association if there exists an unmeasured covariate with a relative risk association at least as large as the E-value. E-values were computed for significant associations and were NA for nonsignificant findings (eMethods and eResults in the Supplement).

^c^ Rituximab was used as the reference group.

^d^ For each relapse outcome, we applied 2 adjustments, outcome regression and propensity scores, to derive the doubly robust estimation.

^e^ With rituximab as the reference, a positive difference in the 1-year or 2-year relapse rate or a relative risk (ratio) of non-relapse rates less than 1 would indicate higher relapse probability of natalizumab.

^f^ Dimethyl fumarate was used as the reference group.

^g^ With dimethyl fumarate as the reference, a positive difference in the 1-year or 2-year relapse rate or a relative risk (ratio) of non-relapse rates less than 1 would indicate higher relapse probability of fingolimod.

In the adapted E-value sensitivity analysis^42,43,44,45^ to assess the unmeasured confounding, the E-values* for the DR estimators after adjustment of full EHR features ranged from 1.06 to 1.31 (Table 2; eMethods and eFigure 1 in the Supplement). E-values assess the strength of the unmeasured confounding that would change the direction of association, while E-values* assess the strength of the unmeasured confounding that would negate the significance of the observed associations. In contrast, the association between features and MS relapse was relatively small (eTable 3 in the Supplement [eg, the feature with the largest relative risk was 1.03 in the time-to-relapse analysis]). The relatively moderate to large E-values indicated that the conclusion had a moderate to large tolerance to unmeasured confounding. When compared with the DR estimators adjusting for registry-derived features, the full EHR feature analysis reduced potential unmeasured confounding, particularly for the treatment association with the 2-year relapse rate (eMethods and eResults in the Supplement).

In examining the adaptive LASSO-selected high-dimensional features (ie, features with nonzero coefficients), we observed confounders with clinical relevance to both treatment assignment and relapse outcomes (eg, female; normalized MS *ICD* code in the overall period preceding treatment initiation; normalized MS *CUI* in the preceding 3 months; normalized corticosteroid prescription in the preceding 3 months; *CUI* C0029134 [“optic neuritis”] in the preceding 3 months; *CUI* C0311394 [“difficulty in walking”] and *CUI* C0518214 [“perceived quality of life”] in the overall period preceding treatment initiation) (eTable 3 in the Supplement). Further, some LASSO-selected features were only associated with treatment assignment given their nonzero coefficient in the propensity score model but zero coefficient in the outcome regression models (eg, health care utilization in the preceding 3 months or normalized MRI use), while other features were only associated with relapse outcomes given their nonzero coefficient for one of the outcome regression models but zero coefficient in the propensity score model (eg, number of relapses in prior 1 year and prior 2 years or *CUI* C0202205 [“oligoclonal band measurement”]). Interestingly, this data-driven approach identified features seemingly not associated with either the treatment assignment or relapse outcomes that were nevertheless important to adjust (eg, *CPT* pulmonary procedures, *CPT* cytopathology procedures, or *CUI* C0242350 [“erectile dysfunction”]). eTable 5 in the Supplement lists the descriptions of EHR features selected by the adaptive LASSO for all analyses.

### Dimethyl Fumarate vs Fingolimod

**Figure 3.**
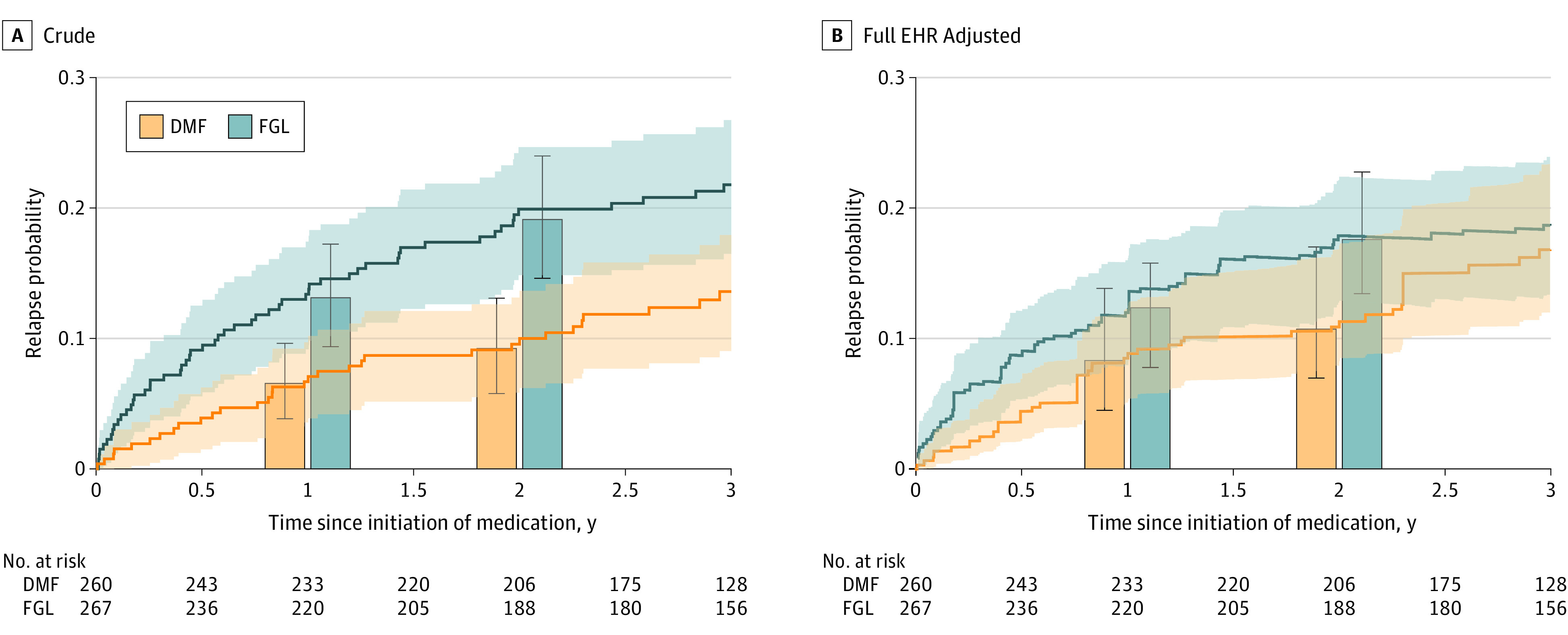
Multiple Sclerosis Relapse for Patients Treated With Dimethyl Fumarate (DMF) or Fingolimod (FGL) Based on Registry-Annotated Treatment Groups The lines depict the cumulative incidence curves for time to relapse, obtained from (A) Kaplan-Meier for crude estimation and (B) mean estimated relapse probability based on doubly robust (DR) estimation with adjustment of high-dimensional full electronic health record (EHR) features in addition to expert-defined features (based on clinical knowledge). The bars indicate the relapse rates at 1 year and 2 years since treatment initiation according to crude and DR analyses. Shaded areas and error bars indicate the 95% CIs. We reported the results based on the DR estimation in Table 2.

In the primary analysis of the registry-annotated treatment groups with adjustment for the high-dimensional set of full EHR covariates that includes expert-defined features (eTable 4 in the Supplement), we found no significant difference in 1-year relapse rate (0.028 [95% CI, –0.031 to 0.084]) or time to relapse (0.957 [95% CI, 0.884-1.035]) (Table 2). Although the fingolimod group had a higher 2-year relapse rate than the dimethyl fumarate group with a DR estimate of 0.071 (95% CI, 0.008-0.128), the adjusted *P* value of .08 did not meet the threshold for multiple testing (Table 2; Figure 3). We again found reduction in confounding by the full EHR analysis (eMethods and eResults in the Supplement).

### Supplementary Results

We reported findings of the unadjusted analysis, registry-derived analysis, and expert-defined analysis as benchmark comparison and results using the EHR RxNorm–identified treatment groups (natalizumab vs rituximab: eMethods and eResults and eTable 6 in the Supplement; dimethyl fumarate vs fingolimod: eMethods and eResults and eTable 7 in the Supplement), as well as the largely consistent, time-adjusted analysis findings (eMethods and eResults and eTable 8 in the Supplement) and DMT adherence rate (eFigure 2 in the Supplement).

## Discussion

In this study using real-world observational data, we compared 2 pairs of commonly prescribed DMTs for their association with MS relapse outcomes using the research registry–annotated treatment groups as the primary analysis and EHR RxNorm–identified treatment groups as exploratory analyses. To balance patient characteristics for each pair of DMT comparisons, we extensively adjusted for confounding factors using full EHR feature sets and a data-driven approach for feature selection using LASSO. In the standard-efficacy DMT comparison, we found no significant difference in all 3 relapse outcomes (ie, 1-year relapse rate, 2-year relapse rate, and time to relapse) between dimethyl fumarate and fingolimod after adjusting for full EHR covariates (including expert-defined features). In the higher-efficacy DMT comparison, adjustment for the high-dimensional full EHR covariates resulted in consistently lower relapse rates in favor of rituximab relative to natalizumab for all 3 relapse outcomes, whereas adjustment for registry-derived features yielded nonsignificant findings and adjustment for the expert-defined (without full EHR) confounders yielded inconsistent results in favor of rituximab (ie, only the relative risk of relapse). This inconsistency could be partially due to the omission of important confounders such as narrative mentions of quality of life, difficulty walking, and optic neuritis, as well as certain unexpected features that were associated with both relapse risk and treatment assignment (eg, pulmonary procedures) . Finally, the consistent results between the research registry–annotated and EHR RxNorm–identified treatment groups supported the generalizability of the approach.

To fill the knowledge gap due to the absence of randomized clinical trials comparing commonly prescribed DMTs, this study contributes to the growing literature leveraging real-world evidence. Two important aspects differentiate our study from prior observational studies of comparative effectiveness of DMTs in MS. First, building on our group’s prior works integrating EHR and registry data,^25,26,27,28^ this study incorporated within the high-dimensional models the additional confounders from the EHR data that are not typically available in registry data. Our comparison of natalizumab and rituximab particularly illustrated the utility of incorporating the high-dimensional EHR features to balance patient characteristics. Specifically, the analyses adjusting for the registry-derived features did not yield significant differences, while analyses adjusting only for the expert-defined features (without full EHR features) mirrored the conflicting results from prior literature^18,19,20^ and yielded inconsistent findings among the 3 relapse outcomes. In contrast, analyses adjusting for full EHR features in addition to expert-defined features consistently demonstrated better relapse outcomes in favor of rituximab over natalizumab. This finding suggests the potential advantage associated with incorporating detailed patient-level EHR data to adjust for confounding biases more comprehensively. Importantly, we found that the high-dimensional EHR features reduced unmeasured confounding when compared with registry-derived features only. Second, this study included parallel analyses between registry-annotated and EHR RxNorm–identified treatment groups. The comparable results from parallel approaches of patient identification and treatment assignment supported the feasibility of conducting similar comparative effectiveness DMT analysis using EHR data for treatment assignment. While well-annotated registry data are limited because of the labor-intensive nature of registry studies, EHR data are often more readily available.

### Limitations

Our study has some limitations. First, the potential confounding by indication may not be fully corrected even after adjusting for full EHR features via DR estimation. After propensity score balancing,^34,36^ certain differences in the baseline characteristics between the treatment groups persisted. This finding is expected because of the need to simultaneously balance multiple features. The DR method additionally corrected for confounding bias by modeling how the confounders were associated with the outcome. Our approach of DR estimation modeling with adjustment of high-dimensional covariates from the EHR data, including narrative features from clinical notes (that may capture information related to clinical decisions and care received outside of the system), is an important mitigating step to address this limitation common to all observational studies, including many published studies to date. Given the extensive balancing tests performed, multiple tests could produce small *P* values for some features. Even randomized clinical trials have occasional imbalances between treatment groups. Second, there is the potential limitation of generalizability, as the study participants in the CLIMB registry were patients in a single academic MS clinic in the US with relatively low overall relapse rates. Given the unique opportunity to analyze registry and EHR data in parallel, the motivation of this study is to develop methods that would enable other centers to replicate our findings and generalize the EHR-based approach. Third, the study did not consider treatment adherence and addressed only the intent to treat (ie, is the decision to start one DMT associated with a lower relapse rate when compared with the other DMT?). To fully examine the continuous contribution of DMT beyond the treatment decision, structured nested models^48^ could be explored, although there are notable methodological challenges to adjust high-dimensional confounders in the already complex structured nested models. Fourth, data leakage due to care received outside the health care system was a potential limitation, although it was common to all EHR research in countries without a unified health care and EHR system. As MGB represents the largest health care system in its region and encompasses multiple affiliated hospitals while the patients with MS in the CLIMB cohort receive their MS care exclusively and other medical care predominantly in MGB, the possibility of EHR data leakage in this study is low. Furthermore, we leveraged clinician notes and natural language processing to further capture clinical conditions evaluated and managed outside of MGB.

## Conclusions

This comparative effectiveness study based on observational data comparing 2 pairs of commonly prescribed DMTs found no significant difference in relapse between dimethyl fumarate and fingolimod and consistently better relapse outcomes in favor of rituximab over natalizumab. These findings based on high-dimensional modeling that incorporates EHR data address knowledge gaps in MS treatment guidance where randomized clinical trials are unavailable and likely infeasible. Future studies examining outcomes of long-term disability and “no evidence of disease activity” are warranted. Our approach is potentially applicable to the broader treatment comparison field based on real-world evidence, particularly when research registry data are lacking while EHR data are readily available.

## Notes

Supplement.
eMethods and eResults.
**eTable 1.** Feature Description for the Registry-Annotated Treatment Groups of MS Patients: LASSO-Selected Confounders From the Full EHR Feature Analysis Comparing Natalizumab (NTZ) vs Rituximab (RTX) **eTable 2.** Feature Description for the Registry-Annotated Treatment Groups of MS Patients: LASSO-Selected Confounders From the Full EHR Feature Analysis Comparing Dimethyl Fumarate (DMF) vs Fingolimod (FGL) **eTable 3.** Standardized Coefficient of Features Selected by Adaptive LASSO for the Natalizumab (NTZ) vs Rituximab (RTX) Comparison **eTable 4.** Standardized Coefficient of Features Selected by Adaptive LASSO for the Dimethyl Fumarate (DMF) vs Fingolimod (FGL) Comparison **eTable 5.** Description for Electronic Health Record Features Selected by Adaptive LASSO **eTable 6.** Estimated Comparative Treatment Outcome of Natalizumab (NTZ) vs Rituximab (RTX) Using Rituximab as the Reference Group **eTable 7.** Estimated Comparative Treatment Outcome of Dimethyl Fumarate (DMF) vs Fingolimod (FGL) Using Dimethyl Fumarate (DMF) as the Reference Group **eTable 8.** Time-Adjusted or Matched Estimated Comparative Treatment Outcomes of Natalizumab (NTZ) vs Rituximab (RTX) Using RTX as the Reference Group and Dimethyl Fumarate (DMF) vs Fingolimod (FGL) Using DMF as the Reference Group Based on Registry-Annotated Treatment Groups **eFigure 1.** Evaluation of Unmeasured Confounding in the Registry-Derived Feature Analyses That Were Explained in the “Full EHR” Feature Analyses of the Comparative Treatment Outcomes **eFigure 2.** Disease-Modifying Therapy Adherence Rate for the Registry-Annotated Treatment Groups in the Study
eReferences.
