# Supplementary material for: Comparison of Dimethyl Fumarate vs Fingolimod and Rituximab vs Natalizumab for Treatment of Multiple Sclerosis
Source: JAMA Netw Open. 2021 Nov 16;4(11):e2134627. doi: 10.1001/jamanetworkopen.2021.34627 (PMC8596196; doi:10.1001/jamanetworkopen.2021.34627)
Supplement: Supplement. — eMethods and eResults. eTable 1. Feature Description for the Registry-Annotated Treatment Groups of MS Patients: LASSO-Selected Confounders From the Full EHR Feature Analysis Comparing Natalizumab (NTZ) vs Rituximab (RTX) eTable 2. Feature Description for the Registry-Annotated Treatment Groups of MS Patients: LASSO-Selected Confounders From the Full EHR Feature Analysis Comparing Dimethyl Fumarate (DMF) vs Fingolimod (FGL) eTable 3. Standardized Coefficient of Features Selected by Adaptive LASSO for the Natalizumab (NTZ) vs Rituximab (RTX) Comparison eTable 4. Standardized Coefficient of Features Selected by Adaptive LASSO for the Dimethyl Fumarate (DMF) vs Fingolimod (FGL) Comparison eTable 5. Description for Electronic Health Record Features Selected by Adaptive LASSO eTable 6. Estimated Comparative Treatment Outcome of Natalizumab (NTZ) vs Rituximab (RTX) Using Rituximab as the Reference Group eTable 7. Estimated Comparative Treatment Outcome of Dimethyl Fumarate (DMF) vs Fingolimod (FGL) Using Dimethyl Fumarate (DMF) as the Reference Group eTable 8. Time-Adjusted or Matched Estimated Comparative Treatment Outcomes of Natalizumab (NTZ) vs Rituximab (RTX) Using RTX as the Reference Group and Dimethyl Fumarate (DMF) vs Fingolimod (FGL) Using DMF as the Reference Group Based on Registry-Annotated Treatment Groups eFigure 1. Evaluation of Unmeasured Confounding in the Registry-Derived Feature Analyses That Were Explained in the “Full EHR” Feature Analyses of the Comparative Treatment Outcomes eFigure 2. Disease-Modifying Therapy Adherence Rate for the Registry-Annotated Treatment Groups in the Study eReferences. [file jamanetwopen-e2134627-s001.pdf]

Supplementary Online Content

Hou J, Kim N, Cai T, et al. Comparison of dimethyl fumarate vs fingolimod and rituximab vs natalizumab for treatment of multiple sclerosis. *JAMA Netw Open.* 2021;4(11):e2134627. doi:10.1001/jamanetworkopen.2021.34627

eMethods and eResults.
eTable 1. Feature Description for the Registry-Annotated Treatment Groups of MS Patients: LASSO-Selected Confounders From the Full EHR Feature Analysis Comparing Natalizumab (NTZ) vs Rituximab (RTX)
eTable 2. Feature Description for the Registry-Annotated Treatment Groups of MS Patients: LASSO-Selected Confounders From the Full EHR Analysis Comparing Dimethyl Fumarate (DMF) vs Fingolimod (FGL)
eTable 3. Standardized Coefficient of Features Selected by Adaptive LASSO for the Natalizumab (NTZ) vs Rituximab (RTX) Comparison
eTable 4. Standardized Coefficient of Features Selected by Adaptive LASSO for the Dimethyl Fumarate (DMF) vs Fingolimod (FGL) Comparison
eTable 5. Description for Electronic Health Record Features Selected by Adaptive LASSO
eTable 6. Estimated Comparative Treatment Outcome of Natalizumab (NTZ) vs Rituximab (RTX) Using Rituximab as the Reference Group
eTable 7. Estimated Comparative Treatment Outcome of Dimethyl Fumarate (DMF) vs Fingolimod (FGL) Using Dimethyl Fumarate (DMF) as the Reference Group
eTable 8. Time-Adjusted or Matched Estimated Comparative Treatment Outcomes of Natalizumab (NTZ) vs Rituximab (RTX) Using RTX as the Reference Group and Dimethyl Fumarate (DMF) vs Fingolimod (FGL) Using DMF as the Reference Group Based on Registry-Annotated Treatment Groups
eFigure 1. Evaluation of Unmeasured Confounding in the Registry-Derived Feature Analyses That Were Explained in the "Full EHR" Feature Analyses of the Comparative Treatment Outcomes
eFigure 2. Disease-Modifying Therapy Adherence Rate for the Registry-Annotated Treatment Groups in the Study
eReferences.

This supplementary material has been provided by the authors to give readers additional information about their work.

© 2021 Hou J et al. *JAMA Network Open.*

eMethods and eResults

1. Natalizumab (NTZ) versus Rituximab (RTX)

1.1 Multiple Testing Adjustment.

To adjust for the multiple testing (difference in 1-year relapse rate, difference in 2-year relapse rate and relative risks of 2-year relapse from time-to relapse), we bootstrapped the distribution of the maximal absolute value of the three Wald test statistics (**Table 2**). In each of the 10000 bootstrap repeats, we used the same bootstrap sample to calculate the three bootstrap estimates for difference in 1-year relapse rate, difference in 2-year relapse rate and relative risks of 2-year relapse, which preserved the correlation of the estimates. We standardized each of the three bootstrap estimates, turning them into bootstrap Wald statistics. We took the maximum of the absolute value for each bootstrap Wald statistics and used it to empirically evaluate the distribution of the maximal absolute value of the three Wald test statistics.

After the multiple testing adjustment, the p-values were .02 for difference in 1-year relapse rate, .004 for difference in 2-year relapse rate and .01 for relative risks of 2-year relapse with RTX as the reference (**Table 2**).

1.2 Adapted E-value Sensitivity Analysis to Assess Unmeasured Confounding.

We adapted the E-value sensitivity analysis[1], which was developed for discrete feature space with small number of states, to high dimensional setting with many continuous elements (**Table 2**). Specifically, we performed the following steps: (1) stratify the feature space by the propensity score (PS) and outcome regression (OR) model predictions; (2) calculate the E-value with covariate stratification for the DR estimation and its 95% confidence interval obtained via the bootstrap. A key quantity in the E-value analysis is the **bounding factor**, which quantifies the unmeasured confounding under a hypothetical true outcome model. The bounding factor can also quantify the reduction in unmeasured confounding between two nested models assuming the larger model is the true model. We adopted this bounding factor approach to illustrate the degree to which adjustment for additional EHR factors reduces the confounding biases compared to the analysis solely based on registry data. Given the limited number of observed relapse events, we stratified the feature space into 8 strata for difference in 1-year or 2-year relapse rate (the three-way interaction of high/low OR prediction for NTZ relapse, high/low OR prediction for RTX relapse, and high/low propensity for RTX) and 4 strata for relative risks of 2-year non-relapse (the two-way interaction of high/low OR prediction for relapse risk and high/low propensity for RTX). Interactions were multiplicative. High propensity for RTX was the same as low propensity for NTZ.

We summarized the E-values in **Table 2**. For difference in 1-year relapse rate, the residual unmeasured confounding would negate the significance of the association if its bounding factor were greater than 1.13 and would change the direction of the association if its bounding factor were greater than 1.50. For difference in 2-year relapse rate, the unmeasured confounding would negate the significance of the association if its bounding factor were greater than 1.31 and would change the direction of the association if its bounding factor were greater than 2.26. For the relative risk of 2-year non-relapse (from time-to-relapse analysis), the unmeasured confounding would negate the significance of the association if its bounding factor were greater than 1.06 and would change the direction of the association if its bounding factor were greater than 1.11. Given that the maximal relative risks among full EHR features associated with the time-to-relapse outcome was 1.03 (**eTable 3**), the E-values were relatively moderate to large, indicating that the conclusion had a moderate to large tolerance to unmeasured confounding.

To evaluate the unmeasured confounding in the registry-derived features that was explained by the high-dimensional full EHR features that included expert-defined features, we calculated the bounding factor for the registry-derived analysis[1], treating hypothetically the fitted OR and PS model from the *full EHR feature analysis* as the underlying true models (**eFigure 1**). The OR and PS model predictions from the full EHR feature analysis were the hypothetical unmeasured confounders in the calculation. Similar to E-value sensitivity analysis for the "full EHR" analysis, we discretized the feature space into 10 by 10 strata according to the OR and PS model predictions from the registry-derived analysis (see **Supplementary Material 1.4**). We also

© 2021 Hou J et al. *JAMA Network Open.*

discretized each hypothetical unmeasured confounders to 10 levels. Within each stratum, we calculated the bounding factor using the OR and PS model predictions from the "full EHR" analysis as the true models for outcomes and exposures given the covariates and unmeasured confounders. We visualized the bounding factors as heat maps. The bounding factors for empty strata were set as one. The average bounding factor was calculated as the mean over the bounding factors of all strata proportional to their sizes. Higher bounding factor values indicated greater extent of confounding unexplained by registry features but explained by high-dimensional EHR features. Here, the average bounding factors were 1.06 for 1-year relapse rate, 7.38 for 2-year relapse rate, and 1.04 for time-to-relapse. These results indicated that unmeasured residual confounding in registry-derived analysis was explained by the "full EHR analysis", particularly for the 2-year relapse rate.

1.3 Expert-defined Analysis.

In the *expert-defined analysis* using registry-annotated treatment groups with RTX as the reference (**eTable 6**), after adjusting for covariates based on clinical knowledge, we found no significant difference in the 1-year relapse rate or 2-year relapse rate between the NTZ group and the RTX group after correction for multiple testing, while patients in the NTZ group had a significantly lower relative risk of 2-year non-relapse rate 0.926 (95% CI = 0.870–0.980, p-value .01 and adjusted p-value .04). The difference in results between the "full EHR analysis" and "expert-defined analysis" suggested that the high-dimensional full EHR feature set captured additional, important confounders not among the low-dimensional expert-defined covariates.

The analyses using the EHR RxNorm-identified treatment groups demonstrated results consistent with the primary analysis based on registry-annotated treatment groups (**eTable 6**). After adjusting for expert-defined set of covariates in the *expert-defined analysis*, the NTZ group had a marginally higher 1-year relapse rate than the RTX group, but there was no significant difference in 2-year relapse rate or time-to-relapse between the two groups after correction for multiple testing. As with the registry-annotated groups, in the *full EHR analysis*, RTX had lower relapse rate than NTZ for all three relapse outcomes after correction for multiple testing: 1-year relapse rate (DR estimate=0.091; 95% CI=0.046-0.138, p-value <.001 and adjusted p-value .001), 2-year relapse rate (DR estimate=0.138; 95% CI =0.058–0.188, p-value <.001 and adjusted p-value .001), and time-to-relapse (DR estimate=0.910; 95% CI=0.856–0.984, p-value .01 and adjusted p-value .04).

1.4 Benchmark Analysis.

For benchmark, we performed: (1) the crude analysis without any adjustment; (2) the registry-derived analysis with feature exclusively from the CLIMB registry (gender, race, age at MS diagnosis, follow-up duration, disease duration, prior relapse within 12 months and 24 months) (**eTable 6**). In the unadjusted / crude analysis, RTX was associated lower relapse when compared to NTZ: 1-year relapse rate (DR estimate=0.086; 95% CI=0.025-0.150, p-value .01 and adjusted p-value .01), 2-year relapse rate (DR estimate=0.171; 95% CI=0.101–0.248, p-value <.001 and adjusted p-value <.001), and time-to-relapse (DR estimate=0.863; 95% CI=0.821–0.906, p-value <.001 and adjusted p-value <.001). In the registry-derived analysis, RTX was not significantly different from NTZ after correction for multiple testing: 1-year relapse rate (DR estimate=0.119; 95% CI=0.015-0.222, p-value .02 and adjusted p-value .08), 2-year relapse rate (DR estimate=0.113; 95% CI=0.003–0.217, p-value .03 and adjusted p-value .07), and time-to-relapse (DR estimate=0.930; 95% CI=0.873–0.997, p-value .04 and adjusted p-value .10).

1.5 Time-adjusted Analysis.

Patients in the NTZ and RTX groups initiated treatment during 2006-2016. To adjust for potential temporal effect during the 10-year span, we conducted a time-adjusted analysis (**eTable 8**). In the analysis, we included the following features: OR and PS model predictions from the original analysis, time indicators for DMT initiation in 2006-2008, 2009-2011 and 2012-2016, interaction between time indicators and the features selected in the OR and PS model by adaptive Lasso. As with the original analysis, we found that RTX was associated with lower relapse rate when compared to NTZ for the 1-year relapse rate (DR estimate=0.074; 95% CI=0.019-0.139, p-value .01 and adjusted p-value .05) and 2-year relapse rate (DR estimate=0.132; 95% CI=0.055–0.236, p-value <.001 and adjusted p-value .01), and nearly significant for the time-to-relapse (DR estimate=0.906;

© 2021 Hou J et al. *JAMA Network Open.*

95% CI=0.840–0.999, p-value .05 and adjusted p-value .07). Point estimates were similar to "Full EHR" analysis. The elevated adjusted p-values might be due to the additional variability from temporal adjustments.

2. Dimethyl Fumarate (DMF) versus Fingolimod (FGL)

2.1 Multiple Testing Adjustment.

Using the same multiple testing adjustment (see **Supplementary Material 1.1**), we calculated the adjusted p-values after accounting for multiple testing (**Table 2**). The adjusted p-values were .38 for difference in 1-year relapse rate, .08 for difference in 2-year relapse rate and .50 for relative risks of 2-year relapse. Thus, there was no significant difference in the 1-year relapse rate, 2-year relapse rate, or time-to-relapse between the DMF and the FGL group.

2.2 Adapted E-value Sensitivity Analysis to Assess Unmeasured Confounding.

To evaluate the unmeasured confounding in the registry-derived features that was explained by the high-dimensional "full EHR" features that included expert-defined features, we calculated the bounding factor[1], treating the OR and PS model predictions from the *full EHR feature analysis* as the hypothetical unmeasured confounders in the registry-derived analysis (**eFigure 1**). Here, the average bounding factors were 4.66 for 1-year relapse rate, 7.35 for 2-year relapse rate, and 1.10 for time-to-relapse. Thus, unmeasured confounding in registry-derived analysis was explained by the "full EHR" analysis, particularly for 1-year and 2-year relapse rates.

2.3 Expert-defined Analysis.

In the *expert-defined analysis* using registry-annotated treatment groups with DMF as the reference (**eTable 7**), after adjusting for the covariates based on clinical knowledge, we found no significant difference in the 1-year relapse rate, 2-year relapse rate, or time-to-relapse between the DMF and the FGL group.

The analyses using the EHR RxNorm-identified treatment groups likewise did not find significant difference in the 1-year relapse rate, 2-year relapse rate, or time-to-relapse between the DMF and FGL group in either the *expert-defined analysis or* the high-dimensional *full EHR analysis* (**eTable 7**).

2.4 Benchmark Analysis.

For benchmark, we again performed: (1) the crude analysis without any adjustment; (2) the registry-derived analysis with feature exclusively from the registry (gender, race, age at MS diagnosis, follow-up duration, disease duration, prior relapse within 12 months and 24 months) (**eTable 7**). In the unadjusted / crude analysis, DMF was associated with the lower relapse when compared to FGL: 1-year relapse rate (DR estimate=0.062; 95% CI = 0.008-0.111, p-value .02 and adjusted p-value .05), 2-year relapse rate (DR estimate=0.099; 95% CI=0.036–0.158, p-value <.001 and adjusted p-value .006), and time-to-relapse (DR estimate=0.926; 95% CI=0.878–0.978, p-value <.001 and adjusted p-value .01). In the registry-derived analysis, we found no significant difference in the 1-year relapse rate, 2-year relapse rate, or time-to-relapse between the DMF and the FGL group after correction for multiple testing.

2.5 Time-adjusted Analysis.

Patients in the FGL and DMF groups initiated treatment during 2010-2016 and 2013-2016, respectively. To adjust for potential temporal effect during the nonoverlapping time span, we conducted a time-matched analysis (**eTable 8**). In this analysis, we excluded FGL patients who initiated DMT during 2010-2012 to match the observation window 2013-2016 for DMF since FGL received FDA approval in 2010 whereas DMF received FDA approval in 2013. We included the following features: OR and PS model predictions from the original analysis and the features selected in the OR and PS model by adaptive Lasso. As with the original analysis, we found no significant difference in the 1-year relapse rate, 2-year relapse rate, or time-to-relapse between the DMF and the FGL group.

© 2021 Hou J et al. *JAMA Network Open.*

eTable 1. Feature Description for the Registry-Annotated Treatment Groups [1] of MS Patients: LASSO-Selected Confounders [2] From the Full EHR Feature Analysis [3] Comparing Natalizumab (NTZ) vs Rituximab (RTX)

Feature [3-5]	Overall	Observed [6]			After IPW Balancing [6]		
		NTZ	RTX	p-val	NTZ	RTX	p-val
Female, No. (%)	243 (76.2)	160 (78.4)	83 (72.2)	.26	154 (75.6)	80 (69.5)	.22
Normalized MS ICD code overall	0.5 (0.2)	0.5 (0.2)	0.5 (0.2)	.47	0.5 (0.2)	0.5 (0.2)	.52
Normalized MS CUI code within 3 months	0.08 (0.09)	0.07 (0.07)	0.1 (0.1)	<.001	0.07 (0.07)	0.09 (0.09)	.04
Normalized corticosteroids use within 3 months	0.02 (0.03)	0.02 (0.03)	0.02 (0.04)	.06	0.02 (0.03)	0.02 (0.04)	.04
CPT pulmonary overall	0.1 (0.4)	0.08 (0.3)	0.2 (0.5)	.02	0.08 (0.3)	0.1 (0.4)	.16
CUI C0518214 [quality of life] overall	0.1 (0.4)	0.10 (0.3)	0.2 (0.5)	.01	0.09 (0.3)	0.2 (0.4)	.1
CUI C0311394 [difficulty walking] overall	0.2 (0.4)	0.1 (0.3)	0.3 (0.6)	<.001	0.2 (0.4)	0.3 (0.5)	.09
CUI C0029134 [optic neuritis] within 3 months	0.1 (0.4)	0.2 (0.4)	0.1 (0.4)	.13	0.2 (0.4)	0.1 (0.4)	.29

Note:

1. The registry-annotated cohort included 204 patients in the NTZ arm and 115 in the RTZ arm.
2. The table presented only the confounders, defined as the features affecting *both* treatment assignment and MS relapse. We identified confounders as the features with nonzero coefficients for propensity score model and for at least one of the outcome regression models from the adaptive LASSO.
3. Full EHR feature analysis included full EHR features in addition to expert-defined features. We constructed features according to multiple timeframes, including *3 months* or *6 months* or *overall* period prior to treatment initiation.
4. Please refer to **eTable 5** for full description of narrative features (*i.e.*, concept unique identifiers, *CUI*).
5. For binary/categorical features, we reported counts (proportion). For numerical features, we reported mean (standard deviation).
6. We calculated the p-values for balance from the Pearson's chi-square test for binary/categorical features and Wilcoxon rank sum test for numerical features. We calculated the p-values for post-inverse probability weighting (IPW) balancing from the weighted chi-square test for binary/categorical features and weighted Wilcoxon rank sum test for numerical features.

© 2021 Hou J et al. *JAMA Network Open.*

eTable 2. Feature Description for the Registry-Annotated Treatment Groups [1] of MS Patients: LASSO-Selected Confounders [2] From the Full EHR Feature Analysis [3] Comparing Dimethyl Fumarate (DMF) vs Fingolimod (FGL)

Feature [3-5]	Overall	Observed [6]			After IPW Balancing [6]		
		DMF	FGL	p-val	DMF	FGL	p-val
Normalized MS CUI code within 3 months	0.1 (0.1)	0.1 (0.1)	0.1 (0.1)	.002	0.1 (0.1)	0.1 (0.1)	.18
Normalized corticosteroids use within 3 months	0.0 (0.0)	0.0 (0.0)	0.0 (0.0)	.001	0.0 (0.0)	0.0 (0.0)	.63
Normalized MRI overall	0.2 (0.1)	0.2 (0.1)	0.2 (0.1)	.03	0.2 (0.1)	0.2 (0.1)	.89
Normalized MRI within 6 months	0.1 (0.1)	0.1 (0.1)	0.1 (0.1)	.06	0.1 (0.1)	0.1 (0.1)	.17
PheCode 418 [nonspecific chest pain] overall	0.1 (0.4)	0.2 (0.5)	0.1 (0.3)	.11	0.2 (0.5)	0.1 (0.3)	.07
PheCode 427.5 [arrhythmia (cardiac) NOS] overall	0.1 (0.3)	0.1 (0.3)	0.1 (0.3)	.47	0.1 (0.3)	0.1 (0.3)	.33
PheCode 599.4 [urinary incontinence] overall	0.1 (0.4)	0.2 (0.5)	0.1 (0.3)	.02	0.2 (0.5)	0.1 (0.3)	.11
PheCode 722.6 [degeneration of intervertebral disc] overall	0.1 (0.4)	0.2 (0.5)	0.1 (0.3)	.01	0.2 (0.5)	0.1 (0.3)	.13
CUI C0701466 [Solumedrol] overall	1.1 (1.0)	1.0 (1.0)	1.2 (1.0)	.03	1.1 (1.0)	1.2 (0.9)	.25
CUI C0563322 [intravenous steroids] overall	0.2 (0.4)	0.1 (0.3)	0.3 (0.5)	<.001	0.1 (0.3)	0.2 (0.5)	.05
CUI C0311394 [difficulty walking] overall	0.2 (0.4)	0.2 (0.5)	0.1 (0.3)	.35	0.2 (0.5)	0.1 (0.3)	.26
CUI C0751967 [relapsing remitting multiple sclerosis] overall	0.3 (0.6)	0.4 (0.6)	0.2 (0.5)	.02	0.3 (0.6)	0.2 (0.5)	.29
CUI C0271051 [macular edema] overall	0.3 (0.4)	0.1 (0.3)	0.4 (0.4)	<.001	0.2 (0.4)	0.3 (0.4)	<.001
CUI C0241158 [skin scar] overall	0.1 (0.3)	0.1 (0.4)	0.1 (0.3)	.03	0.1 (0.4)	0.1 (0.3)	.09
CPT MRI brain within 3 months	0.5 (0.5)	0.4 (0.5)	0.5 (0.5)	.16	0.4 (0.5)	0.4 (0.5)	.80
CUI C0717787 [Glatiramer] within 3 months	0.1 (0.3)	0.1 (0.4)	0.1 (0.3)	.50	0.2 (0.5)	0.1 (0.3)	.33
CUI C0023524 [progressive multifocal leukoencephalopathy] within 3 months	0.1 (0.3)	0.1 (0.3)	0.1 (0.3)	.23	0.1 (0.3)	0.1 (0.3)	.76

© 2021 Hou J et al. *JAMA Network Open.*

CUI C0003241 [antibodies] within 3 months	0.2 (0.4)	0.1 (0.4)	0.2 (0.4)	.002	0.2 (0.5)	0.2 (0.4)	.16
CUI C0016382 [flushing] within 3 months	0.2 (0.4)	0.2 (0.4)	0.1 (0.4)	.01	0.2 (0.4)	0.1 (0.4)	.05
CUI C0027497 [nausea or feeling queasy] within 3 months	0.1 (0.4)	0.2 (0.4)	0.1 (0.3)	.34	0.2 (0.6)	0.1 (0.4)	.49
CUI C0271051 [macular edema] within 3 months	0.2 (0.3)	0.0 (0.1)	0.3 (0.4)	<.001	0.1 (0.2)	0.2 (0.4)	<.001

Note:

1. The registry-annotated cohort included 260 patients in the DMF arm and 267 in the FGL arm.
2. The table presented only the confounders, defined as the features affecting *both* treatment assignment and MS relapse. We identified confounders as the features with nonzero coefficients for propensity score model and for at least one of the outcome regression models from the adaptive LASSO.
3. Full EHR feature analysis included full EHR features in addition to expert-defined features. We constructed features according to multiple timeframes, including *3 months* or *6 months* or *overall* period prior to treatment initiation.
4. Please refer to **eTable 5** for full description of narrative features (*i.e.,* concept unique identifiers, *CUI*) as well as *PheCodes*.
5. For binary/categorical features, we reported counts (proportion). For numerical features, we reported mean (standard deviation).
6. We calculated the p-values for balance from the Pearson's chi-square test for binary/categorical features and Wilcoxon rank sum test for numerical features. We calculated the p-values for post-inverse probability weighting (IPW) balancing from the weighted chi-square test for binary/categorical features and weighted Wilcoxon rank sum test for numerical features.

© 2021 Hou J et al. *JAMA Network Open.*

eTable 3. Standardized Coefficient [1] of Features Selected by Adaptive LASSO [2] for the Natalizumab (NTZ) vs Rituximab (RTX) Comparison

	PS	OR 1-year relapse		OR 2-year relapse		Time to relapse (RR) [6]
		NTZ	RTX	NTZ	RTX	
**Features affecting both treatment assignment and relapse outcome (confounders)** [3, 7]						
Female	-0.2	0.06	0	0.04	0	1
Normalized MS ICD code overall	0.04	0	0.94	0	0	1
Normalized MS CUI code within 3 months	0.11	-0.04	-0.88	-0.17	-0.01	0.99
Normalized corticosteroids use within 3 months	-0.06	-0.33	-0.16	-0.39	0	1
CPT pulmonary overall	0.41	0.33	0	0.46	0	1
CUI C0518214 [quality of life] overall	0.23	0.46	0	0.24	0	1
CUI C0311394 [difficulty walking] overall	0.27	0	0	-0.09	0	1
CUI C0029134 [optic neuritis] within 3 months	-0.05	0	-0.01	0	-0.22	1
**Features affecting only treatment assignment** [4, 7]						
Health care uses within 3 months	-0.89	0	0	0	0	1
Normalized MRI overall	-0.24	0	0	0	0	1
Normalized MRI within 6 months	0.34	0	0	0	0	1
Normalized Hospitalization overall	-0.42	0	0	0	0	1
CPT cytopathology overall	0.63	0	0	0	0	1
CUI C0277556 [recurrent disease] overall	-0.28	0	0	0	0	1
CUI C0021400 [influenza] overall	-0.09	0	0	0	0	1
CUI C0004134 [ataxia] overall	0.18	0	0	0	0	1
CUI C1699926 [Fingolimod] overall	0.15	0	0	0	0	1
CPT cytopathology within 3 months	1.24	0	0	0	0	1
CUI C0277556 [recurrent disease] within 3 months	-0.12	0	0	0	0	1
CUI C0021747 [interferon] within 3 months	-0.26	0	0	0	0	1
CUI C0004134 [ataxia] within 3 months	0.22	0	0	0	0	1
CUI C0242350 [erectile dysfunction] within 3 months	0.44	0	0	0	0	1
CUI C0037763 [spasm or muscle cramp] within 3 months	0.08	0	0	0	0	1
CUI C0023524 [progressive multifocal leukoencephalopathy] within 3 months	-0.2	0	0	0	0	1
CUI C0003241 [antibodies] within 3 months	-0.27	0	0	0	0	1
CUI C0030252 [palpitation] within 3 months	0.11	0	0	0	0	1
CUI C0027497 [nausea or feeling queasy] within 3 months	0.29	0	0	0	0	1
CUI C0012569 [diplopia or double vision] within 3 months	-0.14	0	0	0	0	1
CUI C0033684 [protein] within 3 months	0.09	0	0	0	0	1
**Features affecting only relapse outcome in both the NTZ and RTX treatment group** [5, 7]						
No. of relapses within prior 1 year	0	0	0.85	0.41	0.75	1.01
No. of relapses within prior 2 years	0	0.49	0.9	0.27	1.08	1.03
CPT other procedures overall	0	-0.4	0.43	-0.22	0.18	1
CUI C0202205 [oligoclonal bands measurement] overall	0	0.01	1.04	0.06	0.15	1
**Features affecting only relapse outcome in the NTZ treatment group** [5, 7]						
Normalized MS ICD code within 3 months	0	0.16	0	0	0	1

© 2021 Hou J et al. *JAMA Network Open.*

PheCode 743.9 [osteopenia or other disorder of bone and cartilage] overall	0	0.23	0	0	0	1
PheCode 599.5 [frequency of urination and polyuria] overall	0	-0.36	0	0	0	1
CUI C0023524 [progressive multifocal leukoencephalopathy] overall	0	0	0	-0.32	0	1
CPT Office/other outpatient services within 3 months	0	0	0	0.24	0	1
CUI C0020538 [hypertension] within 3 months	0	0	0	-0.17	0	1
CUI C0015672 [fatigue or decreased energy] within 3 months	0	0	0	-0.39	0	1
CUI C0549206 [pregnancy (not delivered)] within 3 months	0	0	0	-0.16	0	1
CUI C0079595 [diagnostic imaging technique] within 3 months	0	-0.06	0	-0.11	0	1
**Features affecting only relapse outcome in the RTX treatment group** [5, 7]						
CUI C0563322 [intravenous steroids] overall	0	0	0	0	0.88	1
CPT microbiology within 3 months	0	0	0.26	0	0.14	1
CUI C0423551 [sensory symptoms] within 3 months	0	0	0.18	0	0.25	1

Note:

1. The standardized coefficient is the product of the model coefficient from adaptive LASSO and the standard deviation of the feature. By standardizing the coefficients, we made the coefficient invariant to scaling of features. Thus, the coefficients for features of different frequencies became comparable.
2. The classification of the features was based on nonzero coefficients from adaptive LASSO. Standardized coefficients between -0.01 and 0.01 were considered as zero.
3. Features affecting both the treatment assignment and the MS relapse were the confounders presented in **eTable 1**. These features had nonzero propensity score (*PS*) coefficient and at least one nonzero outcome regression (*OR*) coefficient.
4. Features affecting only the treatment assignment had nonzero PS coefficient but no nonzero OR coefficient.
5. Features affecting only relapse outcomes had at least one nonzero OR coefficient but no nonzero PS coefficient. We further divided these features (or risk factors for relapse) into three categories: those affect MS relapse in NTZ arm, those affect MS relapse in RTX arm and those affect MS relapse in both arms according to their selection in the specific OR models.
6. The relative risk (RR) of the feature in the time-to-relapse analysis was calculated as the exponential of the standardized coefficient.
7. Please refer to **eTable 5** for full description of narrative features (i.e., concept unique identifiers, *CUI*) as well as *PheCodes*.

© 2021 Hou J et al. *JAMA Network Open.*

eTable 4. Standardized Coefficient [1] of Features Selected by Adaptive LASSO [2] for the Dimethyl Fumarate (DMF) vs Fingolimod (FGL) Comparison

	PS	OR 1-year relapse		OR 2-year relapse		Time to relapse (RR) [6]
		DMF	FGL	DMF	FGL	
**Features affecting both treatment assignment and relapse outcome** [3, 7]						
Normalized MS CUI code within 3 months	-0.13	0	0	0	-0.3	0.99
Normalized corticosteroids use within 3 months	0.09	-0.13	-0.18	0	-0.37	0.98
Normalized MRI overall	-0.31	0	-0.19	0	-0.23	0.99
Normalized MRI within 6 months	0.22	0	0	0	0.02	1
PheCode 418 [nonspecific chest pain] overall	-0.02	-0.13	0	0	0	1
PheCode 427.5 [arrhythmia (cardiac) NOS] overall	0.02	-0.37	0	-0.28	0	1
PheCode 599.4 [urinary incontinence] overall	-0.12	-0.07	0	0	0	1
PheCode 722.6 [degeneration of intervertebral disc] overall	-0.02	0	-0.18	0	-0.66	1
CUI C0701466 [Solumedrol] overall	0.26	0.13	0	0.02	0	1.01
CUI C0563322 [intravenous steroids] overall	0.21	0	0.01	0	0.13	1
CUI C0311394 [difficulty walking] overall	-0.02	-0.76	-0.01	0	0	1
CUI C0751967 [relapsing remitting multiple sclerosis] overall	-0.14	0	0	0	-0.06	1
CUI C0271051 [macular edema] overall	0.57	0.72	0	0	0	1
CUI C0241158 [skin scar] overall	-0.23	0	0	0.14	0	1
CPT MRI brain within 3 months	-0.14	0	0	0.29	0	1
CUI C0717787 [Glatiramer] within 3 months	-0.13	-0.02	0	0	0	1
CUI C0023524 [progressive multifocal leukoencephalopathy] within 3 months	-0.12	0.42	0.19	0.13	0	1.02
CUI C0003241 [antibodies] within 3 months	0.17	0	0	0	0.18	1
CUI C0016382 [flushing] within 3 months	-0.04	0	0.07	0	0.09	1
CUI C0027497 [nausea or feeling queasy] within 3 months	-0.19	-0.36	0	0	0	1
CUI C0271051 [macular edema] within 3 months	1.16	-0.85	0	-0.12	0	1
**Features affecting only treatment assignment** [4, 7]						
Female	-0.12	0	0	0	0	1
Follow up duration, years	-0.54	0	0	0	0	1
Normalized Hospitalization overall	-0.03	0	0	0	0	1
PheCode 743.9 [osteopenia or other disorder of bone and cartilage] overall	-0.06	0	0	0	0	1
CUI C0025260 [memory] overall	-0.08	0	0	0	0	1
CUI C0026838 [spasticity or muscle spasticity] overall	-0.29	0	0	0	0	1
CUI C0042866 [vitamin D] overall	-0.04	0	0	0	0	1
CUI C0038999 [swelling] overall	-0.18	0	0	0	0	1
CUI C0079595 [diagnostic imaging technique] overall	-0.28	0	0	0	0	1
CPT organ or disease-oriented panels within 3 months	0.28	0	0	0	0	1
CPT cardiovascular within 3 months	0.75	0	0	0	0	1
CUI C0025260 [memory] within 3 months	-0.2	0	0	0	0	1
CUI C1304680 [attack (or sudden onset of symptom)] within 3 months	0.3	0	0	0	0	1

© 2021 Hou J et al. *JAMA Network Open.*

CUI C0032743 [positron-emission tomography] within 3 months	0.13	0	0	0	0	1
CUI C0042866 [vitamin D] within 3 months	-0.09	0	0	0	0	1
**Features affecting only relapse outcome in both the DMF and FGL treatment group** [5, 7]						
Normalized MS ICD code overall	0	0.06	0.05	0.06	0.05	1.02
Normalized corticosteroids use overall	0	0.68	0	0	-0.18	1
No. of relapses within prior 1 year	0	0.21	0.01	0.27	0	1
No. of relapses within prior 2 years	0	0.97	0	0	0	1.02
CUI C0277556 [recurrent disease] overall	0	0.02	0.06	0.38	0	1.02
CUI C0023524 [progressive multifocal leukoencephalopathy] overall	0	0.28	0.04	0.35	0.01	1.01
CUI C0021368 [inflammation] overall	-0.01	1.04	-0.15	0.19	-0.1	1
CUI C0233844 [clumsiness] overall	0	0.34	0.09	0.19	0.15	1.01
CUI C0277556 [recurrent disease] within 3 months	0	0.23	0.02	0.24	0	1
CUI C0020538 [hypertension] within 3 months	0	-0.19	-0.02	-0.05	-0.41	1
CUI C0015672 [fatigue or decreased energy] within 3 months	-0.01	-0.01	0	0	0.29	1
CUI C0543488 [interest or concern] within 3 months	0	0	-0.4	-0.17	-0.42	0.99
CUI C0549206 [pregnancy (not delivered)] within 3 months	0	0.19	0	0	0	1.01
CUI C0004268 [attention or concentration] within 3 months	0	-0.14	0	0	-0.06	1
**Features affecting only relapse outcome in the FGL treatment group** [5, 7]						
Non-Hispanic European	0	0	0.02	0	0	0.99
Health care uses within 3 months	0	0	0.01	0	0.13	1.01
PheCode 272.1 [hyperlipidemia]overall	0	0	-0.08	0	-0.02	1
CPT cytopathology overall	0	0	0	0	-0.3	1
CUI C0038317 [steroid] overall	0	0	0.29	0	0.73	1
CUI C0393022 [Rituximab] overall	0	0	-0.04	0	-0.2	0.99
CUI C0684336 [impairment] overall	0	0	0.29	0	0.12	1
CUI C0225326 [fiber] overall	0	0	0	0	-0.11	1
CUI C0231170 [disability] within 3 months	0	0	0	0	0.3	1
CUI C0026838 [spasticity or muscle spasticity] within 3 mo	0	0	0	0	0.17	1
CUI C0021747 [interferon] within 3 months	0	0	0	0	-0.17	0.99
CUI C0004134 [ataxia] within 3 months	0	0	-0.19	0	-0.18	1
CUI C0009443 [common cold] within 3 months	0	0	0.08	0	0.37	1
CUI C0029134 [optic neuritis] within 3 months	0	0	0.07	0	0.14	1
**Features affecting only relapse outcome in the DMF treatment group** [5]						
CUI C0202205 [oligoclonal bands measurement] overall	0	0	0	0.07	0	1.01
CUI C0701466 [Solumedrol] within 3 months	0	0.34	0	0	0	1.01
CUI C0033684 [protein] within 3 months	0	0	0	-0.24	0	1

© 2021 Hou J et al. *JAMA Network Open.*

Note:

1. The standardized coefficient is the product of the model coefficient from adaptive LASSO and the standard deviation of the feature. By standardizing the coefficients, we made the coefficient invariant to scaling of features. Thus, the coefficients for features of different frequencies became comparable.
2. The classification of the features was based on nonzero coefficients from adaptive LASSO. Standardized coefficients between -0.01 and 0.01 were considered as zero.
3. Features affecting both the treatment assignment and the MS relapse were the confounders presented in **eTable 2**. These features had nonzero propensity score (*PS*) coefficient and at least one nonzero outcome regression (*OR*) coefficient.
4. Features affecting only the treatment assignment had nonzero PS coefficient but no nonzero OR coefficient.
5. Features affecting only relapse outcomes had at least one nonzero OR coefficient but no nonzero PS coefficient. We further divided these features (or risk factors for relapse) into three categories: those affect MS relapse in DMF arm, those affect MS relapse in FGL arm and those affect MS relapse in both arms according to their selection in the specific OR models.
6. The relative risk (RR) of the feature in the time-to-relapse analysis was calculated as the exponential of the standardized coefficient.
7. Please refer to **eTable 5** for description of narrative features (i.e., concept unique identifiers, *CUI*) as well as *PheCodes*.

© 2021 Hou J et al. *JAMA Network Open.*

eTable 5. Description for Electronic Health Record Features Selected by Adaptive LASSO

**Code [1, 2]**	**Description**	**Higher-efficacy DMT Analysis [3]**	**Standard-efficacy DMT Analysis [3]**
PheCode 272.1	Hyperlipidemia	N	Y
PheCode 418	Nonspecific chest pain	N	Y
PheCode 427.5	Arrhythmia (cardiac) NOS	N	Y
PheCode 599.4	Urinary incontinence	N	Y
PheCode 599.5	Frequency of urination and polyuria	Y	N
PheCode 722.6	Degeneration of intervertebral disc	N	Y
PheCode 743.9	Osteopenia or other disorder of bone and cartilage	Y	Y
CUI C0003241	Antibodies	Y	Y
CUI C0004134	Ataxia	Y	Y
CUI C0004268	Attention or concentration	N	Y
CUI C0009443	Common cold	N	Y
CUI C0012569	Diplopia or double vision	Y	N
CUI C0015672	Fatigue or decreased energy	Y	Y
CUI C0016382	Flushing	N	Y
CUI C0020538	Hypertension	Y	Y
CUI C0021368	Inflammation	N	Y
CUI C0021400	Influenza	Y	N
CUI C0021747	Interferon	Y	Y
CUI C0023524	Progressive multifocal leukoencephalopathy	Y	Y
CUI C0025260	Memory	N	Y
CUI C0026838	Spasticity or muscle spasticity	N	Y
CUI C0027497	Nausea or feeling queasy	Y	Y
CUI C0029134	Optic neuritis	Y	Y
CUI C0030252	Palpitation	Y	N
CUI C0032743	Positron-emission tomography	N	Y
CUI C0033684	Protein	Y	Y
CUI C0037763	Spasm or muscle cramp	Y	N
CUI C0038317	Steroid	N	Y
CUI C0038999	Swelling	N	Y
CUI C0042866	Vitamin D	N	Y
CUI C0079595	Diagnostic imaging technique	Y	Y
CUI C0202205	Oligoclonal bands measurement	Y	Y
CUI C0225326	Fiber	N	Y
CUI C0231170	Disability	N	Y
CUI C0233844	Clumsiness	N	Y
CUI C0241158	Skin scar	N	Y
CUI C0242350	Erectile dysfunction	Y	N
CUI C0271051	Macular edema	N	Y
CUI C0277556	Recurrent disease	Y	Y
CUI C0311394	Difficulty walking	Y	Y

© 2021 Hou J et al. *JAMA Network Open.*

CUI C0393022	Rituximab	N	Y
CUI C0423551	Sensory symptoms	Y	N
CUI C0518214	Quality of life	Y	N
CUI C0543488	Interest or concern	N	Y
CUI C0549206	Pregnancy (not delivered)	Y	Y
CUI C0563322	Intravenous steroids	Y	Y
CUI C0684336	Impairment	N	Y
CUI C0701466	Solumedrol	N	Y
CUI C0717787	Glatiramer	N	Y
CUI C0751967	Relapsing remitting multiple sclerosis	N	Y
CUI C1304680	Attack (or sudden onset of symptom)	N	Y
CUI C1699926	Fingolimod	Y	N

Note:

1. Each *PheCode* or "Phenotype" code represented a single clinically informative condition. We used a published classification system that consolidated and mapped multiple related *ICD* (9 or 10) codes to a single PheCode to represent each unique clinical condition.
2. Concept unique identifiers (*CUIs*) indicated clinical terms extracted using natural language processing.
3. Analysis columns indicated the analyses in which the variables were selected by adaptive LASSO. See **eTable 1 and 3** for higher-efficacy DMT analysis, and **eTable 2 and 4** for standard-efficacy DMT analysis.

© 2021 Hou J et al. *JAMA Network Open.*

eTable 6. Estimated Comparative Treatment Outcome of Natalizumab (NTZ) vs Rituximab (RTX) Using Rituximab as the Reference Group

Treatment Group [1]	Feature Set [2]	DR Estimate [3] (95 % CI)	P-value (P-value$_{adj}$) [4]
**Difference in 1-Year Relapse Rate [5]**			
Registry	Crude / Unadjusted	0.086 (0.025,0.150)	.01 (.01)
Registry	Registry-derived	0.119 (0.015, 0.222)	.02 (.08)
Registry	Expert-defined	0.035 (-0.037,0.111)	.32 (.54)
Registry	Full EHR [6]	0.080 (0.013,0.137)	.02 (.02)
EHR RxNorm	Expert-defined	0.074 (0.008,0.124)	.03 (.05)
EHR RxNorm	Full EHR	0.091 (0.046,0.138)	<.001 (.001)
**Difference in 2-Year Relapse Rate [5]**			
Registry	Crude / Unadjusted	0.171 (0.101,0.248)	<.001 (<.001)
Registry	Registry-derived	0.113 (0.003, 0.217)	.04 (.12)
Registry	Expert-defined	0.099 (0.013,0.182)	.03 (.07)
Registry	Full EHR [6]	0.132 (0.043,0.189)	.004 (.004)
EHR RxNorm	Expert-defined	0.070 (-0.017,0.159)	.10 (.21)
EHR RxNorm	Full EHR	0.138 (0.058,0.188)	<.001 (.001)
**Relative Risk of 2-Year Non-relapse Rate (from Time-to-Relapse Analysis) [5]**			
Registry	Crude / Unadjusted	0.863 (0.821,0.906)	<.001 (<.001)
Registry	Registry-derived	0.930 (0.873, 0.997)	.04 (.10)
Registry	Expert-defined	0.926 (0.870,0.980)	.01 (.04)
Registry	Full EHR [6]	0.903 (0.822,0.944)	<.001 (.01)
EHR RxNorm	Expert-defined	0.944 (0.896,1.001)	.06 (.13)
EHR RxNorm	Full EHR	0.910 (0.856,0.984)	.01 (.04)

Note:

1. Patients were assigned to treatment groups either according to the CLIMB registry annotation (registry) or the RxNorm electronic prescription records in the MGB EHR system (EHR RxNorm).
2. In addition to the crude / unadjusted analysis, covariate-adjusted analyses included the following sets of features: (1) registry-derived, (2) expert-defined, (3) full EHR, which additionally included expert-defined features.
3. For each outcome, we applied two adjustments, outcome regression (*OR*) and propensity scores (*PS*), to derive the doubly robust (*DR*) estimation.
4. P-values were adjusted for multiple testing among the 3 analyses with the same treatment group and feature set.
5. With RTX as reference, a positive difference in the 1-year or 2-year relapse rate or a relative risk (ratio) of non-relapse rates <1 would indicate higher relapse probability of NTZ.
6. The primary analysis findings were highlighted in green and shown in **Table 2**.

© 2021 Hou J et al. *JAMA Network Open.*

eTable 7. Estimated Comparative Treatment Outcome of Dimethyl Fumarate (DMF) vs Fingolimod (FGL) Using Dimethyl Fumarate (DMF) as the Reference Group

Treatment Group [1]	Feature Set [2]	DR Estimate [3] (95 % CI)	P value (P-value$_{adj}$) [4]
**Difference in 1-Year Relapse Rate [5]**			
Registry	Crude / Unadjusted	0.062 (0.008,0.111)	.02 (.05)
Registry	Registry-derived	0.049 (-0.014,0.113)	.13 (.35)
Registry	Expert-defined	0.007 (-0.061,0.063)	.87 (.99)
Registry	Full EHR [6]	0.028 (-0.031,0.084)	.38 (.38)
EHR RxNorm	Expert-defined	-0.035 (-0.086,0.021)	.24 (.42)
EHR RxNorm	Full EHR	0.014 (-0.031,0.066)	.56 (.87)
**Difference in 2-Year Relapse Rate [5]**			
Registry	Crude / Unadjusted	0.099 (0.036,0.158)	<.001 (.006)
Registry	Registry-derived	0.077 (-0.004,0.153)	.06 (.17)
Registry	Expert-defined	0.026 (-0.047,0.092)	.53 (.78)
Registry	Full EHR [6]	0.071 (0.008,0.128)	.03 (.08)
EHR RxNorm	Expert-defined	-0.013 (-0.075,0.048)	.73 (.93)
EHR RxNorm	Full EHR	0.051 (0.000,0.15)	.5 (.16)
**Relative Risk of 2-Year Non-relapse Rate (from Time-to-Relapse Analysis) [5]**			
Registry	Crude / Unadjusted	0.926 (0.878,0.978)	<.001 (.01)
Registry	Registry-derived	1.049 (0.993,1.108)	.09 (.25)
Registry	Expert-defined	0.985 (0.929,1.044)	.60 (.87)
Registry	Full EHR [6]	0.957 (0.884,1.035)	.28 (.50)
EHR RxNorm	Expert-defined	0.991 (0.943,1.047)	.79 (.98)
EHR RxNorm	Full EHR	0.937 (0.859,1.021)	.14 (.35)

Note:

1. Patients were assigned to treatment groups either according to the CLIMB registry annotation (registry) or the RxNorm electronic prescription records in the MGB EHR system (EHR RxNorm).
2. In addition to the crude / unadjusted analysis, covariate-adjusted analyses included the following sets of features: (1) registry-derived, (2) expert-defined, (3) full EHR, which additionally included expert-defined features.
3. For each outcome, we applied two adjustments, outcome regression (*OR*) and propensity scores (*PS*), to derive the doubly robust (*DR*) estimation.
4. P-values were adjusted for multiple testing among the 3 analyses with the same treatment group and feature set.
5. With DMF as reference, a positive difference in the 1-year or 2-year relapse rate or a relative risk (ratio) of non-relapse rates <1 would both indicate higher relapse probability of FGL.
6. The primary analysis findings were highlighted in green and shown in **Table 2**.

© 2021 Hou J et al. *JAMA Network Open.*

eTable 8. Time-Adjusted or Matched [1] Estimated Comparative Treatment Outcomes of Natalizumab (NTZ) vs Rituximab (RTX) Using RTX as the Reference Group and Dimethyl Fumarate (DMF) vs Fingolimod (FGL) Using DMF as the Reference Group Based on Registry-Annotated Treatment Groups

**Natalizumab (NTZ) versus Rituximab (RTX)**		
**Outcome** [2]	**Estimate (95% CI)**	**P-value (P-value$_{adj}$)** [5]
Difference in 1-Year Relapse Rate [3]	0.074 (0.019,0.139)	.01 (.05)
Difference in 2-Year Relapse Rate [3]	0.132 (0.055,0.236)	<.001 (.01)
Relative Risk of 2-Year Non-relapse Rate (from Time-to-Relapse Analysis) [3]	0.906 (0.840,0.999)	.05 (.07)
**Dimethyl Fumarate (DMF) versus Fingolimod (FGL)**		
**Outcome** [2]	**Estimate (95% CI)**	**P-value (P-value$_{adj}$)** [5]
Difference in 1-Year Relapse Rate [4]	-0.051 (-0.076,0.003)	.07 (.15)
Difference in 2-Year Relapse Rate [4]	-0.005 (-0.077,0.079)	.85 (.99)
Relative Risk of 2-Year Non-relapse Rate (from Time-to-Relapse Analysis) [4]	1.073 (0.955,1.196)	.21 (.55)

Note:

1. Please refer to Supplementary Material 1.5 and 2.5 for the method of time-adjustment and time-matching.
2. For each outcome, we applied two adjustments, outcome regression (*OR*) and propensity scores (*PS*), to derive the doubly robust (*DR*) estimation.
3. With RTX as reference, a positive difference in the 1-year or 2-year relapse rate or a relative risk (ratio) of non-relapse rates <1 would indicate higher relapse probability of NTZ.
4. With DMF as reference, a positive difference in the 1-year or 2-year relapse rate or a relative risk (ratio) of non-relapse rates <1 would both indicate higher relapse probability of FGL.
5. P-values were adjusted for multiple testing among the 3 analyses for the same treatment groups and feature set.

© 2021 Hou J et al. *JAMA Network Open.*

eFigure 1. Evaluation of Unmeasured Confounding in the Registry-Derived Feature Analyses That Were Explained in the “Full EHR” Feature Analyses of the Comparative Treatment Outcomes.

Top panels reported the Natalizumab (NTZ) versus Rituximab (RTX) comparison with RTX as the reference group based on registry-annotated treatment groups. Bottom panels reported the Dimethyl Fumarate (DMF) versus Fingolimod (FGL) comparison using DMF as the reference group also based on registry-annotated treatment groups. The bounding factors, which quantified the unmeasured confounding under a hypothetical true outcome model[1], were calculated in local stratum defined by the outcome regression (OR) prediction and propensity score (PS) prediction from the registry-derived features analyses. The confounders were the OR prediction and PS prediction from the “full EHR” features analyses. “Full EHR” features also included expert-defined features. Each pixel represented a stratum, and its color illustrated the log bounding factor in the stratum. To interpret the colors, blue colors indicated that the registry-derived analysis overestimated the treatment effect, while red colors indicated that the registry-derived analysis underestimated the treatment effect. The white color denoted empty strata, which contained no patients. Darker color indicated stronger confounding unexplained in the registry-derived analysis yet captured by the “full EHR” feature analysis.

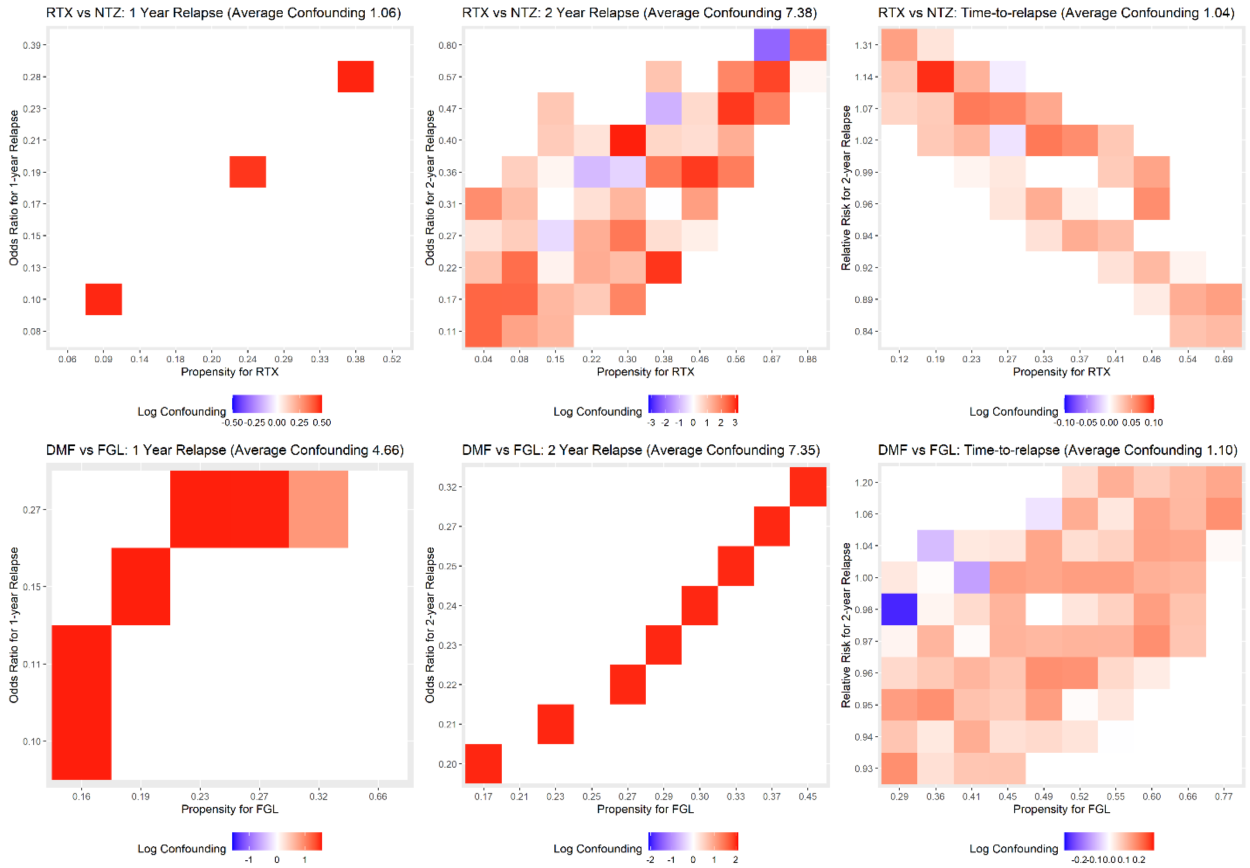

© 2021 Hou J et al. *JAMA Network Open.*

eFigure 2. Disease-Modifying Therapy Adherence Rate for the Registry-Annotated Treatment Groups in the Study.
Natalizumab (NTZ), dimethyl fumarate (DMF) and fingolimod (FGL) groups all had 2-year adherence rate around 60%, while the rituximab (RTX) group had a 2-year adherence rate below 50%.

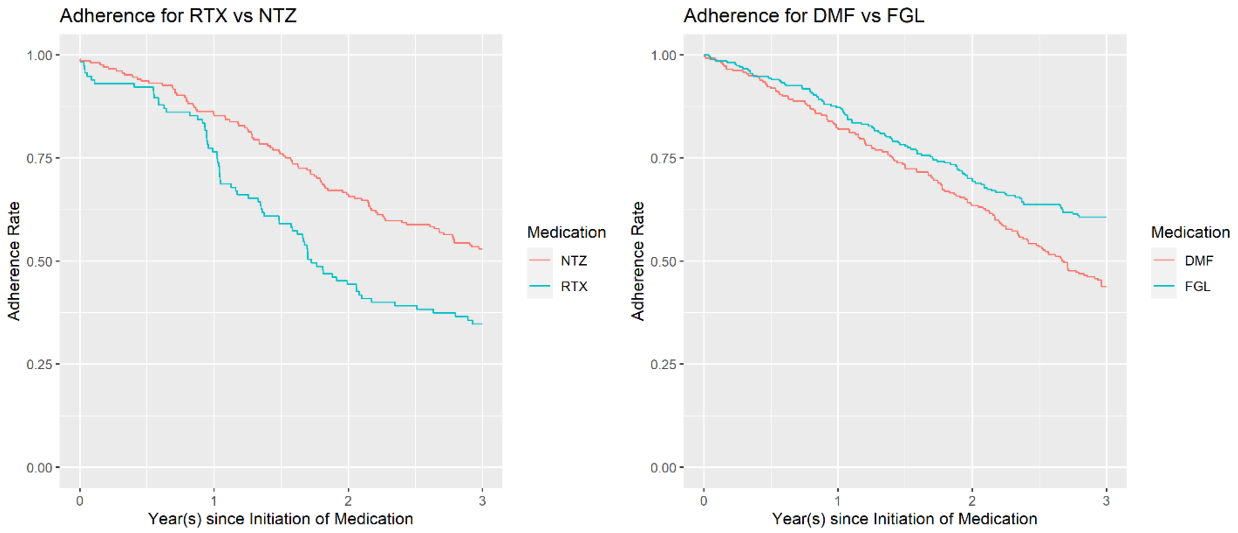

© 2021 Hou J et al. *JAMA Network Open.*

eReferences

1. Ding, P. & VanderWeele, T. J. Sensitivity Analysis Without Assumptions. *Epidemiology* 27, 368–377 (2016).

© 2021 Hou J et al. *JAMA Network Open.*
